# Acquired resistance to anti-PD1 therapy in patients with NSCLC associates with immunosuppressive T cell phenotype

**DOI:** 10.1038/s41467-023-40745-5

**Published:** 2023-08-24

**Authors:** Stefanie Hiltbrunner, Lena Cords, Sabrina Kasser, Sandra N. Freiberger, Susanne Kreutzer, Nora C. Toussaint, Linda Grob, Isabelle Opitz, Michael Messerli, Martin Zoche, Alex Soltermann, Markus Rechsteiner, Maries van den Broek, Bernd Bodenmiller, Alessandra Curioni-Fontecedro

**Affiliations:** 1 Department of Medical Oncology and Hematology, University Hospital Zurich, Zurich, 8091 Switzerland; 2 Comprehensive Cancer Center Zurich, University Hospital Zurich, Zurich, 8091 Switzerland; 3 University of Zurich, Zurich, Switzerland; 4 Department of Quantitative Biomedicine, University of Zurich, Zurich, 8057 Switzerland; 5 Institute of Molecular Health Sciences, ETH Zurich, Zurich, 8049 Switzerland; 6 Life Science Zurich Graduate School, ETH Zurich and University of Zurich, Zurich, Switzerland; 7 Department of Pathology and Molecular Pathology, University Hospital Zurich, 8091 Zurich, Switzerland; 8 Functional Genomics Center Zurich, ETH and University of Zurich, Zurich, 8057 Switzerland; 9 NEXUS Personalized Health Technologies, ETH Zurich, Zurich, 8952 Switzerland; 10 SIB Swiss Institute of Bioinformatics, Lausanne, 1015 Switzerland; 11 Department of Thoracic Surgery, University Hospital Zurich, Zurich, 8091 Switzerland; 12 Department of Nuclear Medicine, University Hospital Zurich, Zurich, 8091 Switzerland; 13 Pathologie Länggasse, Ittigen, 3063 Switzerland; 14 Institute of Experimental Immunology, University of Zurich, Zurich, 8057 Switzerland; 15 Present Address: University of Fribourg, Faculty of Science and Medicine, Fribourg, 1700 Switzerland; 16 Present Address: Clinic of Oncology, Cantonal Hospital Fribourg, Fribourg, 1752 Switzerland

**Keywords:** Translational immunology, Lung cancer, Lymphocyte activation, Cancer immunotherapy

## Abstract

Immune checkpoint inhibitor treatment has the potential to prolong survival in non-small cell lung cancer (NSCLC), however, some of the patients develop resistance following initial response. Here, we analyze the immune phenotype of matching tumor samples from a cohort of NSCLC patients showing good initial response to immune checkpoint inhibitors, followed by acquired resistance at later time points. By using imaging mass cytometry and whole exome and RNA sequencing, we detect two patterns of resistance¨: One group of patients is characterized by reduced numbers of tumor-infiltrating CD8^+^ T cells and reduced expression of PD-L1 after development of resistance, whereas the other group shows high CD8^+^ T cell infiltration and high expression of PD-L1 in addition to markedly elevated expression of other immune-inhibitory molecules. In two cases, we detect downregulation of type I and II IFN pathways following progression to resistance, which could lead to an impaired anti-tumor immune response. This study thus captures the development of immune checkpoint inhibitor resistance as it progresses and deepens our mechanistic understanding of immunotherapy response in NSCLC.

## Abstract (web summary)

Acquired resistance to immune checkpoint inhibitors limits therapeutic success in non-small-cell lung cancer, however, the underpinning immune parameters are largely unknown. Here authors distinguish resistance types based on immune cell infiltration, immune checkpoint molecule and cytokine expression level, using paired samples from patients in the sensitive and in the resistant disease phase.

## Introduction

Lung cancer is a leading cause of cancer-related mortality; patients have a poor prognosis with a median overall survival of 10 to 12 months for advanced stage disease^1^. Treatment of lung cancer has changed dramatically over the last 20 years, with the use of immune checkpoint inhibitors (ICI), which target PD-1 and its ligand PD-L1, leading to improvement in overall survival^2^. Ligation of PD-1 on T cells to PD-L1 expressed on tumor cells suppresses the activity of T cells and leads to a state of exhaustion and dysfunction. Exhausted T cells, however, are not functionally inert. They can be reinvigorated by antibodies that block inhibitory signals such as the PD-1/PD-L1 axis^3–6^. Monoclonal antibodies targeting the PD-1/PD-L1 pathway have good clinical activity in several solid malignancies including non-small cell lung cancer (NSCLC)^7–13^. Nevertheless, only a small proportion of patients develop long-term responses and resistance frequently occurs. In NSCLC, up to 64% of patients who initially respond to ICI develop acquired resistance when an ICI is given as second-line treatment^14^.

Little is known about acquired resistance mechanisms in NSCLC patients, even though many studies have attempted to identify biomarkers predictive of primary unresponsiveness. Primary resistance mechanisms include the absence and loss of tumor antigens^15^, alterations in the MHC processing pathway^16^, low T cell infiltration, enhanced expression of VEGF and immunosuppressive cytokines^17^ and mutations in *STK11* (also known as *LKB1*)^18^. A positive predictive marker is considered to be the PD-L1 expression on tumor cells^13,19,20^, although many patients do not respond to ICI treatment despite having tumors with high PD-L1 expression^21^. Acquired resistance mechanisms have been described in melanoma, lung cancer, and Merkel cell carcinoma patients including upregulation of alternative immune checkpoints, loss of HLA expression, and mutations in *β2-microglobulin* and *JAK1/2*^22–25^. Mutations in *JAK1/2* lead to reduced PD-L1 expression on tumor cells due to impaired IFNγ signaling^26^. When resistance to immunotherapy occurs in NSCLC patients, few therapeutic options are available and no information on the mechanisms of such resistance is known to guide treatment decisions.

The aim of our study is to investigate resistance mechanisms in NSCLC patients who initially responded but became resistant over the course of the treatment with anti-PD-1 checkpoint blockade therapy. We analyze 14 paired tumor samples from seven NSCLC patients prior to therapy and after development of resistance to ICI and investigate the tumor microenvironment using highly-multiplexed imaging mass cytometry (IMC). This allows the simultaneous and deep analysis of multiple features of the tumor microenvironment. From patients with sufficient tissue, we also analyze mutational profiles and gene expression differences between response and resistance. Our study provides deep insight into the acquired resistance mechanisms of NSCLC patients upon anti-PD-1 treatment.

## Results

### Patient history

**Fig. 1:**
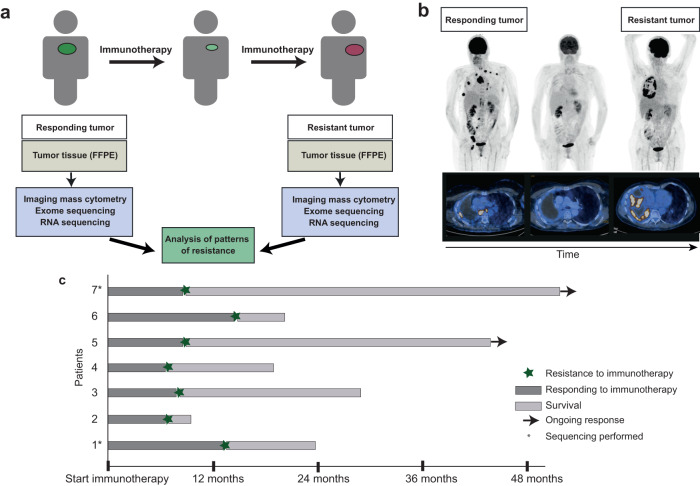
Schematic overview on the cohort of NSCLC patients with acquired resistance to anti-PD-1 therapy. **a** Study scheme involved analysis of tumor biopsies collected before immunotherapy began and after the development of resistance. Whole exome sequencing, RNA sequencing, and imaging mass cytometry analyzes were performed on paraffin-embedded tumors. **b** FDG-PET-CT images of patient #4 before treatment with anti-PD1 (responding tumor), a response, and after development of resistance. **c** Swimmer’s plot illustrating the cohort of the patients included in the study and the length of anti-PD-1 treatment and development of resistance over the course of the disease.

We analyzed 14 paired samples from seven patients with stage IV NSCLC, who initially responded to anti-PD-1 immunotherapy but then developed resistance. All patients had a baseline biopsy or surgery at diagnosis, which yielded the initial tumor samples, and were pre-treated with chemotherapy before immunotherapy (Table 1, Supplementary Table 1). Median overall survival was 567 days from start of immunotherapy; two patients were alive at the time this study was conducted (Fig. 1). We performed whole exome, low coverage whole genome and RNA sequencing of both samples from patients #1 and #7 and targeted sequencing and IMC analysis on paired samples from all seven patients.

**Table 1:** Patient characteristics

Sex	*n*
Female	2
Male	5
**Age, years (range)**	62 (55–67)
**Histology**	
Adenocarcinoma	6
Squamous cell carcinoma	1
**Type of immunotherapy**	
Nivolumab	5
Pembrolizumab	1
Nivolumab + Ipilimumab	1
**Previous therapies**	
Chemotherapy	6
None	1

Patient #1 was diagnosed with stage IIIB lung adenocarcinoma with lymph node involvement but no other distant metastatic lesions. The patient received four cycles of chemotherapy (cisplatin/docetaxel) and sequential radiotherapy of the primary tumor and the affected lymph nodes (66 Gy in 30 fractions, 60 Gy in 30 fractions, respectively). He progressed nine months later and was further treated with 30 cycles of nivolumab. Ten months after immunotherapy started, the patient became resistant to therapy and another sample from the primary tumor was collected.

Patient #2 was diagnosed with stage IV lung adenocarcinoma with metastases in lymph nodes, adrenal gland, and liver. The patient was initially treated with four cycles of chemotherapy (cisplatin/pemetrexed), two cycles of pemetrexed alone, and with sequential radiotherapy (30 Gy in 10 fractions). As the patient progressed under chemotherapy, the treatment was changed to nivolumab/ipilimumab, and thereafter to nivolumab alone due to toxicity of ipilimumab. After the initial response, the patient progressed, and another sample was collected of the primary tumor.

Patient #3 was diagnosed with stage IIIA lung adenocarcinoma. The patient underwent lobectomy with lymphadenectomy followed by three cycles of chemotherapy (cisplatin/gemcitabine). Six months later, a recurrence of stage IV disease was observed, and another biopsy was taken. Due to the presence of a *BRAF* mutation, the patient received a combination therapy of dabrafenib/trametinib until the patient progressed systemically and was diagnosed with brain metastases. The brain metastases were treated with radiotherapy, and immunotherapy was initiated. The patient had received 43 cycles of nivolumab when resistance occurred. Another biopsy from a progressing lesion of the pleura was collected.

Patient #4 was diagnosed with stage IV lung adenosquamous cell carcinoma with lymph node and adrenal gland metastasis. The patient received four cycles of chemotherapy (cisplatin/gemcitabine) and radiotherapy of the adrenal cortex lesion (3 ×12.5 Gy) and a soft tissue metastasis (total 36 Gy). Due to progression, the patient received three cycles of carboplatin/paclitaxel/bevacizumab and palliative radiotherapy. Upon further progression, treatment with nivolumab was started. The patient initially responded to the treatment, but disease progression occurred after 30 cycles. A second biopsy from a progressive lesion of the pleura was collected.

Patient #5 was diagnosed with stage IV lung adenocarcinoma including lymph node involvement and brain metastases. A diagnostic biopsy of the primary tumor before therapy start did not detect any oncogenic alterations using the Oncomine Focus Assay. The patient was treated with immunotherapy (pembrolizumab) and radiotherapy of the brain metastases. Tumor progression occurred after seven months of treatment when a lymph node biopsy was collected.

Patient #6 was diagnosed with stage IV lung adenocarcinoma including brain metastases. The patient was treated with whole brain radiotherapy (30 ×3 Gy) and stereotactic radiotherapy of the primary tumor (15 ×3 Gy). Afterwards, the patient received five cycles of carboplatin/pemetrexed followed by surgery when the initial tumor sample was taken. The patient was treated with three cycles of carboplatin/pemetrexed and due to progression, the treatment was changed to nivolumab. Progression occurred after 21 cycles. At progression, a second tumor sample was collected.

Patient #7 was diagnosed with stage IV lung adenocarcinoma The patient received first-line chemotherapy with platinum and pemetrexed, which was changed to immunotherapy with nivolumab due to progression; the patient partially responded for 15 months. When progression occurred, a biopsy was collected for analysis.

### Genomic profiles of resistant tumors

We were interested in whether newly arising mutations or copy number changes played a role in the acquired resistance to ICI therapy. We therefore performed whole exome sequencing (WES) on the matching tumor samples from two patients, #1 and #7, and compared the nonsynonymous mutations in tumors collected initially and at resistance (Supplementary Fig. 2). In the tumor of patient #1, we detected mutations in tumor suppressor genes such as *MYO18B* and *CTNN2* and in genes related to adhesion, invasiveness, and survival (e.g., *CDH23, HMCN1*, and *OBSCN*) in samples collected at the resistance that were not present initially (Supplementary Data 1). In patient #7, we detected fewer mutations at resistance compared to patient #1, however, mutations in certain tumor suppressor genes such as *NAV3* as well as genes involved in adhesion and invasion (e.g., *ADGRL2, MYEOV*) were detected in the sample at resistance (Supplementary Table 2). We did not see any alterations in genes with known impact on immunotherapy resistance such as *JAK1/2, STAT3*, or *B2M* or in genes encoding PD-1, PD-L1, or CTLA-4 by WES in patient #1 and #7. In addition, no alterations in *JAK1/2, STAT3, PD-1,* and *PD-L1* could be detected in any of the seven patients by FoundationOne sequencing (Supplementary Table 3). Furthermore, we did not detect any mutations in genes previously described to occur in patients with ICI resistance such as *STK11*^18^, *SERPINs*^27^, *PTPN2*^28^, or *APLNR*^29^, and no variation in the expression of *HLA* genes over time was detected. Interestingly, at the time of resistance in patient #1, analysis of copy number variations revealed amplifications in oncogenes like *TERT, KRAS, MET*, and *DDR2*, several SLAM family members, Fc receptors, and in a methyltransferase (*METTL4*), recently described to have an important role in immune suppression^30^ (Supplementary Table 3 and Supplementary Data 2). Patient #7 had no gene amplification in oncogenes but lost annexin-encoding *ANXA10*, which has been associated with cancer aggressiveness^31^ (Supplementary Table 4).

### Downregulation of immune signatures and upregulation of extracellular matrix reorganization

**Fig. 2:**
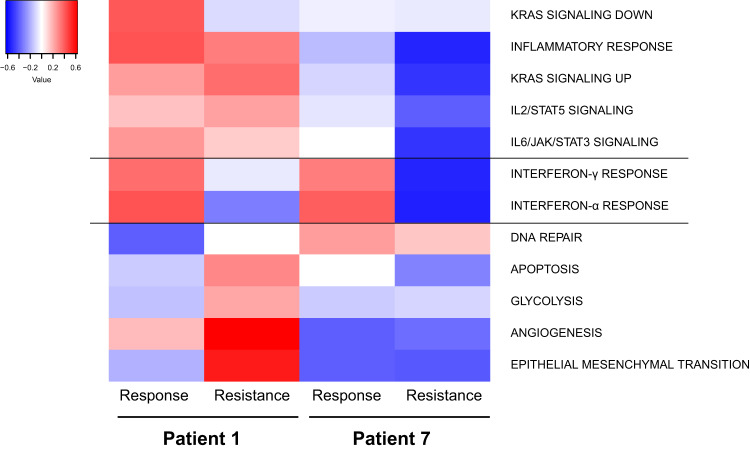
Downregulation of immune signature and upregulation of extracellular matrix reorganization occurs upon resistance development. Heat map of RNA sequencing data showing up- and down-regulated pathways from two patients at response and resistance. The figure is showing gene set enrichment scores according to ref. ^121^.

We performed RNA sequencing of initial tumor samples and samples after resistance was acquired from patients #1 and #7. Interestingly, downregulation of interferon type I and II pathways at the time of resistance was found in both patients (Fig. 2). We computed overlaps with GO terms (Human MSigDB database v2022) of the top 100 up- and downregulated genes at resistance. We detected upregulation in genes involved in extracellular matrix re-organization and collagen network organization in both patients’ samples (Supplementary Tables 5 and 6). This indicates that there is a re-organization of the tumor structure at time of resistance, which could lead to increased aggressiveness and therapy resistance. The 100 most downregulated genes are involved in immune signaling pathways (Supplementary Tables 5 and 6). Specifically, in the tumor from patient #7, genes involved in T cell activation and signaling such as *PTPRC* (which encodes CD45), *LCP2*, *FYB*, *CD3E*, and *LCK,* and in genes involved in immune response and interferon signaling such as *STAT1*, *OAS2*, and *HLA* were downregulated.

### Changes in the immune landscape at the time of resistance revealed by multiplexed imaging mass cytometry

**Fig. 3:**
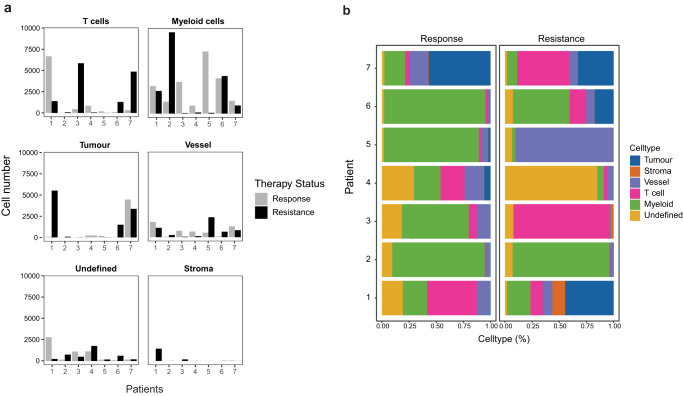
The immune landscape changes at the time of resistance. Overview of different cell types in the tumor detected by IMC analysis at the response and at resistance as shown by **a** total numbers and **b** percentages of indicated cell types. T cells are defined as CD3^+^ cells, myeloid cells as CD11b^+^ cells, tumor cells as pan cytokeratin^+^ cells, endothelial cells as CD31^+^ cells, and fibroblasts as SMA^+^ cells.

To better understand previously described tumor heterogeneity^32^ and changes of cell subtypes upon immunotherapy treatment, samples from each patient were investigated by IMC. A panel of 41 markers was used to characterize tumor, stromal, endothelial, and immune cells (Supplementary Table 7, Supplementary Figs. 3 and 4)^33^. For IMC, samples were stained simultaneously with metal-tagged antibodies and then analyzed resulting in the generation multiplexed image stacks. Image stacks were segmented using a segmentation pipeline to gain information on marker expression on the single-cell level^34,35^. The cells detected consisted mostly of tumor cells (pan cytokeratin^+^), myeloid (CD11b^+^), T (CD3^+^), and endothelial cells (CD31^+^/vWF^+^); fibroblast (SMA^+^)^36^ were less abundant. Clustering based on marker expression resulted in a clear separation of cellular subsets (Supplementary Fig. 5). Two patterns of resistance were detected based on the total cell number changes and percentages (Fig. 3a, b, Supplementary Fig. 6): One patient group was characterized by an increase of T cell infiltration at resistance (patients #3, #6, #7), whereas the other group (patients #1, #2, #4, #5) had similar numbers or reduced numbers of T cell numbers at resistance compared to the initial tumor sample.

### The T cell compartment is altered at the time of resistance

**Fig. 4:**
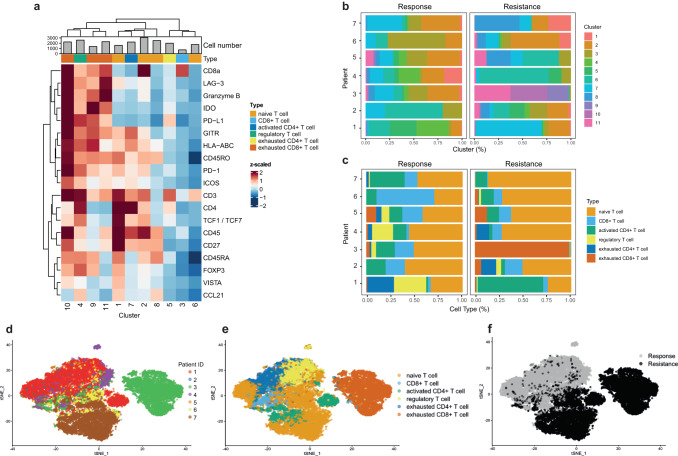
The intratumoral T cell phenotypes are altered upon resistance development. **a** Heat map of T cell marker expression (y-axis) and phenotypic clusters (x-axis) were calculated from IMC raw data. Marker expressions were calculated using the mean intensities for each marker, counts were arcsinh transformed and the clusters were defined using PhenoGraph. The top color bar defines different T cell subsets present in the tumors. **b** Cluster compositions of tumor samples taken from each patient initially and upon resistance development. **c** Percentages of each T cell subtype in tumor samples taken from each patient initially and upon resistance development. **d** *t*-distributed stochastic neighbor embedding (*t*-SNE) map of all T cells color-coded by each patient. **e** t-SNE plot overall T cells color-coded by the different T cell subtype defined in the heat map. **f** t-SNE plot color-coded by responding and resistant tumors.

T cells are important mediators of anti-tumor immunity but are very often unresponsive and upregulate multiple inhibitory immune checkpoint molecules such as PD-1^6^. Checkpoint inhibitor therapy with blocking antibodies reinvigorates the effector functions of T cells^37^. To define T cell phenotypes at the time of resistance, we performed an in-depth analysis of T cell subsets in patient samples before and after the acquisition of resistance to ICI. Unsupervised clustering of cells classified as T cells in IMC images resulted in the identification of 11 distinct T cell clusters (Fig. 4a, b, Supplementary Fig. 7). There are six different T cell subsets: naïve T cell (clusters 1, 2, 6, 8), activated CD4^+^ T cells (cluster 7), CD8^+^ T cells (cluster 3), regulatory T cells (cluster 4), exhausted CD4^+^ T cells (cluster 5), and exhausted CD8^+^ T cells (clusters 9, 10, 11).

Cluster 1 was characterized by expression of T cell markers CD3, CD4, CD27, FoxP3, molecules supporting naïve T cell proliferation such as GITR^38^, or immune inhibitory molecules such as VISTA^39^. TCF-1 was highly expressed on CD4^+^ T cells from cluster 1. TCF-1^+^ CD8^+^ T cells have a high self-renewal capacity and in the absence of antigens can mediate long-term tumor control; TCF-1^-^ CD8^+^ T cells have a shorter survival time^40,41^. In CD4^+^ T cells, TCF-1 expression is important in differentiated T follicular helper cells but not in the Th1 subtype. Moreover, in the absence of TCF-1, germinal center formation and plasma cell development are impaired^42,43^.

Clusters 9 to 11 include cells that are a mixture of different T cell populations including activated but exhausted phenotypes. These clusters are characterized by a high frequency of CD8^+^ T cells expressing immune-inhibitory molecules (e.g. LAG-3, VISTA, PD-L1, PD-1, and IDO), co-stimulatory receptors (e.g. GITR and ICOS), cytotoxic mediators (e.g., Granzyme B), and CD27. CD27 is important for T cell co-stimulation and agonistic targeting, and its expression reduces tumor growth in mice^44,45^. VISTA expression can be induced upon treatment with ICI^46^.

The tumors from patient #3, #4, #5, #6 and #7 taken prior to ICI were enriched in cells from clusters 1, 2, and 3, representing naïve T cells and CD8^+^ T cells, compared to samples taken after resistance developed. In the tumors at resistance, cells from clusters 9, 10, and 11, representing exhausted CD8^+^ T cells, were more abundant than in the initial tumors for those patients (Fig. 4b). TCF-1 was considerably downregulated at resistance (Fig. 4a), indicating a loss of tumor-controlling T cells and the presence of T cells with less self-renewal capacity and a diminished anti-tumor immune response. There were also changes of the percentages of each T cell subtype, with a considerable increase in naive T cells upon resistance development (Fig. 4c).

To further understand expression patterns of each patient during response and resistance, the different cellular subgroups were visualized by t-SNE. This analysis allowed us to characterize cell phenotypes and abundances. Our findings indicate that mechanisms that result in resistance to immunotherapy are heterogeneous. In patient #1, multiple T cell phenotypes were observed in the initial tumor including naïve, regulatory, and exhausted T cells (clusters 4, 5, 6). In the sample from patient #1 collected after resistance developed, there was a reduced number of T cells accompanied by an increase in the tumor and stromal cells; myeloid cell numbers were stable (Fig. 4a). At resistance, cluster 7, representing activated CD4^+^ T cells, constituted of more than 60% of all T cell phenotypes (Fig. 4a, b). Tumor cells expressed PD-L1 in both initial samples and resistant tumors. The tumor sample from patient #2 collected at resistance had a high infiltration of myeloid cells with only a modest change in numbers of T cells, tumor cells, endothelial and stromal cells compared to the initial sample (Fig. 3a, b). The analysis of T cells revealed a reduction in naïve T cells (cluster 6) at resistance and a higher frequency of naïve T cells and exhausted CD4^+^ T cells from clusters 2 and 5. Clusters 8, 10, and 11 were predominant at resistance. These clusters consist of exhausted and also activated CD8^+^ T cells expressing memory, co-stimulatory, and inhibitory markers. Patient #3 presented with an increase in T cell numbers (clusters 9, 10, and 11) at time of resistance but with a loss of myeloid cells. A very distinct exhausted T cell phenotype that has been previously described^47^ was present at resistance in patient #3, clearly separating this patient from the other patients was (Fig. 4d–f). Patient #4 had reduced numbers of T cells at resistance with a shift from high TCF-1^+^, CD27^+^, GITR^+^, PD-1^+^ T cells (clusters 1, 2) toward a more naïve T cell phenotype (cluster 6). This patient also had a complete loss of PD-L1 expression at resistance. In patient #5, clusters 6 and 8 were increased on tumor at resistance, and cluster 2 was reduced. The immunophenotype of cells in the lymph node showed a reduction in Treg (Fig. 4c) and a reduction of PD-L1 (cluster 10 in Figs. 4b and 6b) as well as TCF1 at resistance (Fig. 4b) which is in line with terminally exhausted T cell phenotype. In samples from this patient, we did detect a considerable reduction in myeloid cell numbers and an increase in endothelial cell numbers at resistance (Fig. 3a, b).

In patient #6, there was an increase in endothelial, tumor, and T cell numbers at resistance, but infiltration of myeloid cells was similar in the initial tumor and the sample collected at resistance. At resistance, the tumor was characterized by the loss of naïve CD8^+^ T cells (cluster 3) and a gain of CD8^+^ T cells (clusters 1 and 2) expressing inhibitory markers. At resistance, the tumor sample from patient #7 had an increase of T cell numbers compared to the initial tumor, and there was an increase in T cells with a naïve phenotype at resistance (Fig. 4b). At resistance in this patient, there was a loss of cells from clusters 2 and 7 and gain in clusters 1 and 8.

### The myeloid compartments differ during response and resistance

**Fig. 5:**
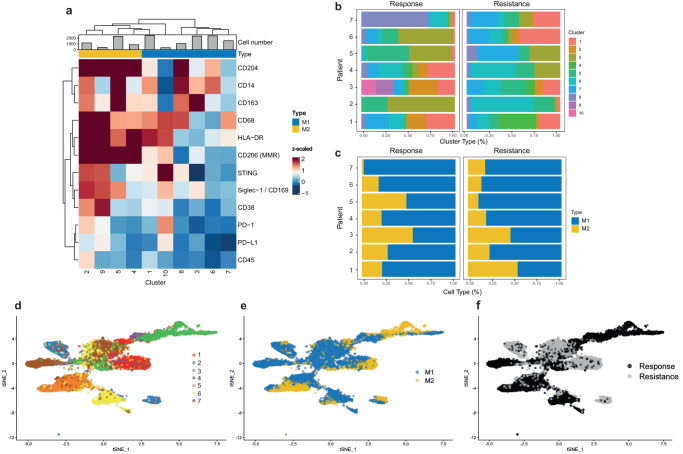
Changes in the myeloid compartment due to resistance detected by IMC differ in each patient. **a** Heat map of myeloid cell marker expression (y-axis) and phenotypic clusters (x-axis) were calculated from IMC raw data. Marker expressions were calculated using the mean intensities for each marker, counts were arcsinh transformed and the clusters were defined using PhenoGraph. The top color bar defines different myeloid cell subsets present in the tumors. **b** Percentages of cells from each patient in indicated clusters at response and at resistance. **c** Percentages of macrophages in M1 (defined as CD169^+^, HLA-DR^+^, STING^+^, and CD38^+^ myeloid cells) and M2 states (define as CD163^+^, CD204^+^, and CD206^+^ myeloid cells) at response and resistance for each patient. **d** t-SNE plot of all macrophages color-coded by patient origin. **e** t-SNE plot color-coded by M1 and M2 macrophage types. **f** t-SNE plot of macrophages color-coded by responding and resistant tumors.

Myeloid cells are important modulators of T cell responses, and treatment with ICI can shift the macrophage phenotype to a more pro-inflammatory phenotype supportive of T cell responses^48^. Thus, we characterized markers of myeloid cells during response and at resistance. Unsupervised clustering of all cells classified as myeloid in IMC images resulted in the identification of 10 distinct clusters (Fig. 5a, b and Supplementary Fig. 8). Cluster 2 had the highest expression and diversity of M1 and M2 macrophage markers, in particular the general macrophage marker CD68, anti-tumor markers CD169, HLA-DR, STING, and CD38, and pro-tumor markers CD163, CD204, and CD206, and immune checkpoint molecules PD-1 and PD-L1. Cluster 4 cells expressed CD204, CD206, and HLA-DR but not STING, CD169, CD38, PD-1, or PD-L1. STING plays an important role in controlling the transcription of different pro-inflammatory genes those involved in type I interferon signaling^49^. Expression of STING in NSCLC is associated with an immune-activating phenotype, which might favor response to ICI treatment^50^. CD38 is expressed by a wide variety of immune cells with multifunctional activities and is involved in adenosine production^51^. Adenosine has immune suppressive effects in the tumor microenvironment, and combination treatment with ICI and CD38 blocking antibody suppressed tumor growth in a T cell-dependent manner^52^.

The pattern of expression of clusters, and the frequency of cluster 4, differed from patient to patient (Fig. 5b). There were no shifts toward an M2 phenotype after resistance developed (Fig. 5c). Myeloid cell phenotypes were differently distributed throughout all samples, and there was almost no overlap in patterns between patients (Fig. 5d–f). In patient #1, loss of cluster 2 and a gain of cluster 4 at resistance was detected, which is in line with a loss in the anti-tumor markers CD169, STING, and CD38 (Fig. 5a). The myeloid cell numbers for this patient were stable (Fig. 3a). In patient #2, we detected a major loss of cluster 3 and a gain in cluster 6 at resistance (Fig. 5a). In both tumors from this patient, over 80% of the cells in the tumor were myeloid cells (Fig. 3a, b), and the myeloid compartment shifted from high expression of CD163 to intermediate expression of CD14 and CD204 at resistance. Expression of the scavenger receptor CD204 is correlated with poor prognosis in solid tumors and with an aggressive tumor phenotype in NSCLC^53^. In patient #3, a loss of clusters 2, 9, and 10 and enhanced expression of cluster 5 were detected at resistance (Fig. 5a); thus, resistance in this patient was associated with reduced expression of HLA-DR, STING, CD169, CD38, PD-1, and PD-L1 and higher expression of CD14 and CD163. In patient #4, the resistant tumor lost enrichment in clusters 1 and 2 and had higher expression of clusters 3, 5, and 6 (Fig. 5a). The myeloid compartment in patient #5 the lymph node biopsy at resistance was enriched in myeloid cells from the tissue of origin (brain). Patient #6 showed a reduction in the percentage of myeloid cells, but stable total numbers (Fig. 3b). In this patient, there was a loss of clusters 3 and 5 and gains in clusters 1 and 7 at resistance, therefore the myeloid compartment changed from high expression of CD206, CD204, CD14, and CD163 to a slight increase in HLA-DR at resistance (Fig. 5a). In patient #7, loss of clusters 3 and 7 and gain of clusters 1 and 8 was detected at resistance, correlating with loss of CD163 and a gain of CD204 with small changes in CD14- and HLA-DR-expressing myeloid cells (Fig. 5a). In summary, there was not a major shift from M1 to M2 phenotypes and the change in macrophage phenotypes upon development of resistance to ICI differed from patient to patient (Fig. 5c–f).

### Two patterns of T cell phenotypes were observed upon resistance development by IHC

**Fig. 6:**
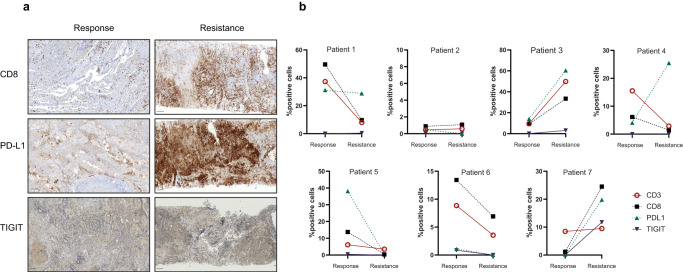
Immunohistochemical analysis reveal two patterns of T cell infiltration during resistance. **a** Representative immunohistochemistry analyzes of immune markers on tumors collected at response and resistance. Scale bar indicates 100 µm. **b** Percentage positivity of specific markers in responsive and resistant tumor samples calculated using QuPath software (one tumor sample per patient was stained for each marker at each time point).

To evaluate the T cell infiltration in responding and resistant tumors, immunohistochemical analyzes for CD3, CD8, PD-L1, and TIGIT were performed on the paired tumor samples, and the percentage of positively stained cells were determined using the digital pathology software QuPath with automated identifiers for each marker (Fig. 6a, b). Here, two patterns of responses were detected at resistance: One patient group lost CD3^+^ and CD8^+^ T cells (patients #1, #4, #5, and #6), and the other group had a higher number of T cells at resistance (patients #3 and #7, Fig. 6b). Patients #3 and #7 also had higher expression of PD-L1 and TIGIT at resistance, whereas in the other patients, with the exception of patient #4, PD-L1 expression dropped or stayed stable at resistance. Patient #4 had high PD-L1 expression at resistance but with low T cell infiltration. The increased expression of inhibitory receptors on T cells is indicative of T cell exhaustion^54^, whereas the expression of TIGIT is characteristic of a dysfunctional T cell subtype that includes regulatory T cells^55^.

## Discussion

Although acquired resistance to ICI therapy occurs in most patients with NSCLC, little is known about the underlying mechanisms, and only one study has examined acquired resistant mechanisms in this setting^23^. Here we applied genomic analyzes and high-dimensional multiplexed imaging to study samples from seven patients with NSCLC taken at prior to ICI and upon the development of resistance. This is the first study to use IMC to analyze NSCLC patient tumors with acquired resistance to anti-PD-1 therapy. We detected the presence of massive T cell infiltrations in the resistant tumor lesions in three out of seven patients. A high number of tumor-infiltrating CD8^+^ T cells was previously shown to correlate with overall survival in NSCLC patients treated with ICI therapy^56^. Our characterization of these infiltrates showed the co-expression of a wide variety of immune checkpoints and immune regulatory enzymes that led to a severely exhausted T cell phenotype with limited effector functions, which could account for the unresponsiveness to ICI therapy despite a broadly distributed T cell presence.

T cell exhaustion due to chronic exposure to antigen results in unresponsiveness, reduced cytotoxic capacity, and co-expression of different inhibitory checkpoint molecules^57,58^. Expression of inhibitory receptors is associated with tumor progression in NSCLC patients treated with anti-PD-1 inhibitors^59^, and resistance to ICI therapy is characterized by the upregulation of alternative immune inhibitory receptors in both mice and humans^24,46^. Targeting some of these immune checkpoints as VISTA, LAG-3, Tim-3, and TIGIT led to additive effects and reactivation of tumor-specific T cells^58,60,61^ and clinical trials are still ongoing (NCT04475523, NCT02812875, (NCT03489369^62–66^. In our cohort, we found an upregulation of Tim-3 at resistance in the tumor specimen of patient #6 (Supplementary Fig. 9), while TIGIT upregulation was found at resistance in patients #3 and #7, suggesting that in these patients, new resistance mechanisms involving these immune checkpoints, occur. The resistant tumors of several patients showed a loss of expression of the transcription factor TCF-1. TCF-1 is important for T cell responses against cancer cells through its effects on the differentiation of mature CD8^+^ T cells. Compared to TCF-1^-^ T cells, T cells expressing TCF-1 have a high self-renewal capacity, even in the absence of antigens, and can mediate long-term tumor control^40,41^ and TCF-1 is necessary for effective anti-tumor responses in preclinical cancer models^67^. Interestingly, melanoma patients with high numbers of TCF-1^+^ CD8^+^ cells have prolonged survival^68^. TCF-1 expression in CD4^+^ T cells is critical in differentiated T follicular helper cells but not in the Th1 subtype. In the absence of TCF-1, germinal center formation and plasma cell development is impaired^42,43^, and recent work demonstrated that T follicular helper cells are needed for an effective CD8^+^ T cell response in tumors^69^. In our cohort of patients, loss of TCF-1 might explain loss of response to ICI, suggesting that interventions in this pathway might provide a route to re-sensitization to ICI.

In the myeloid compartment, we did not detect major differences between responding and resistant tumors. However, this could also be due to the limitation of our antibody panel. Several studies have described different myeloid cell subtypes to be positively or negatively associated with cancer progression. FOLR2^+^ macrophages were positively associated with CD8^+^ effector T cell responses^70^, while a specific subtype of myeloid cells, which differentiate from erythroid progenitors, so-called erythroid differentiated myeloid cells (EDMC), strongly correlated with exhausted CD8^+^ T cells and elevated immune suppression and negatively influenced response to ICI treatment^71^. Patients #1, #4, and #6 received radiotherapy before ICI treatment, this could however influence the TME in the responding tumors. Radiotherapy leads to a local inflammatory response and to recruitment and activation of T cells into the TME^72^. In contrary, immune-related tumor escape mechanisms such as the presence of MDSC and regulatory T cells and the expression of immune checkpoints are not influenced or even enhanced^73^. This could account for the larger proportion of regulatory T cells in patients #1 and #4 and CD8^+^ T cells in patient #6 in the responding tumors (Fig. 4c).

On the genomic level, several aberrations have been linked to resistance to ICI therapy in other tumor types including mutations in the gene encoding β2-microglobulin that lead to impaired recognition of the cancer cells^22,23^. Deletions or mutations in genes that encode proteins involved in antigen processing and presentation machinery can result in immune escape mechanisms and lack of responsive to ICI therapy^16,74,75^. In the tumor samples from the two patients evaluated by WES and RNA sequencing (patients #1 and #7), we did not detect any mutations in *HLA-ABC* or *HLA-DR* genes or in genes involved in the antigen processing and presentation machinery.

Despite the high inter-patient heterogeneity, RNA sequencing of the tumors of patients #1 and #7 revealed the downregulation of type I and II IFN pathways. Links have previously been reported between loss of IFN signaling and enhanced tumorigenesis (reviewed in^76,77^). Tumor and immune cells are in constant cross talk, and interferons are key to this communication. They have stimulatory effects on immune cells^78,79^ and can increase tumor immunogenicity by recruitment of cytotoxic CD8^+^ T cells^80^. Interferons, however, play a dual role in the tumor microenvironment as they also induce the expression of immunosuppressive factors such as IDO and PD-L1 and recruit regulatory T cells^81^, which can facilitate cancer progression and tumor escape^82^. Blocking of PD-L1 on tumor cells leads to the sensitization of tumor cells to interferon-mediated killing. Genetic deficiencies in the IFNγ pathway in the tumor cells hinders activation of the regulatory factor IRF1 and subsequent transcription of the *PD-L1* gene. Blocking antibodies against PD-1/PD-L1 are less effective in patients whose tumor cells express little or no PD-L1. The two patients (#1 and #7) with altered IFN signaling exhibited a very high expression of PD-L1 at time of resistance, which highlights that other factors are important for the lack of response to ICI. The resistance to ICI in these two patients might be explained by a cell-intrinsic suppressive mechanism of PD-L1 on IFN signaling, which protects tumor cells from the cytotoxic effect of IFN^83^.

The RNA sequencing data on both patients (#1 and #7) revealed that the 100 most upregulated genes at the time of resistance were involved in extracellular matrix and collagen reorganization (Supplementary Table 6). Cytoskeleton remodeling and architectural changes in the extracellular matrix influence cell adhesion and immune cell trafficking and migration^84^. Alterations in the collagen structure can also lead to changes in invasion and migration. Collagen degradation by matrix metalloproteases is crucial for enhanced invasion during cancer progression^85,86^. In lung cancer, T cells preferentially migrate in collagen-loose-regions, along collagen type 1 fibrils that are altered in a chemokine-dependent mechanism^87^. However, high collagen densities within the tumor can negatively affect T cell proliferation and induce the expression of regulatory surface markers^88^. Thus, stroma density defines T cell migration capacities and the accumulation of cytotoxic cells within the tissue. Structural changes can lead to immune cell exclusion from certain areas of a tumor^87^. Lung tumors with increased collagen levels were shown to be resistant to anti-PD-1/PD-L1 immunotherapy in a recent study^89^. It was also recently shown that a gene signature that includes several extracellular matrix-associated genes correlates with response to PD-1 therapy in melanoma and bladder cancer^90,91^. Our findings support the use of drugs aiming at targeting the tumor stroma, especially in patients with upregulated genes involved in extracellular matrix deposition and collagen reorganization.

The DNA sequencing data revealed mutations in tumor suppressor genes such as *MYO18B*, *CTNN2*, and *NAV3*^92,93^ and in genes related to adhesion, invasiveness, and survival including *CDH23, HMCN1*, *OBSCN, ADGRL2*, and *MYEOV*^94,95^ in tumor samples at acquired resistance. In patient #1, we also detected amplification of cancer-associated genes *TERT*, *MET*, and *DDR2*. TERT proteins are important for the extension and replenishment of telomeres, which are expressed in embryonic stem cells and become silenced later. The proliferative capacity of cells is limited by the length of the telomeres, which is a crucial tumor suppressor mechanism. Re-expression of *TERT* can lead to immortalization of cancer cells and enhanced survival^96^. MET is a receptor tyrosine kinase for the growth factor HGF, which is involved in cell motility and proliferation^97^ and is overexpressed or dysregulated in several malignancies. c-MET overexpression is correlated with poor prognosis in NSCLC tumors^98^. A mechanism of resistance to ICI therapy could include inhibition of STING induced by *MET* amplifications has been recently described in a preclinical model^99^. In line with these data, we detected a copy number amplification of *MET* in one patient and a loss of cluster 4 which includes STING. This finding supports the mechanism demonstrated in the preclinical model and suggests the importance of molecular testing to be performed at resistance to ICI for therapeutic purposes. Therapies targeting c-MET and PD-L1 using diabodies showed a superior tumor control capacity compared to combination therapy of two antibodies alone in mouse tumor models^100^. A recent phase Ib study (NCT02099058) investigated an anti-c-MET directed antibody-drug conjugate (telisotuzumab vedotin) in combination with nivolumab in advanced NSCLC patients and showed limited anti-tumor activity^101^. Amplifications of *MET* allow targeting with specific tyrosine kinase inhibitors, which has proven successful in lung cancer patients^102^. DDR2 plays an important role in the regulation of cell proliferation, migration, and metastasis^103–105^. Interestingly, DDR2 targeting with the tyrosine kinase inhibitor dasatinib in combination with anti-PD-1 checkpoint inhibitor let to a better response than either single treatment^106^. In addition, DDR2 can modulate AXL expression^107^, while AXL inhibition induces type I interferon and expansion of TCF1^+^CD8^+^ T cells^108^. Thus, amplification of *DDR2* might indirectly lead to a worse response to ICI therapy by downregulating important immune features for response. Such amplifications are not routinely examined in patients upon resistance to immunotherapy. Our data highlight the importance of genomic analysis of tumors at the time of resistance to identify possible targeted treatment options and to guide the development of combinatorial treatments.

In patient #6, we could detect in the responding sample a co-mutation of *LKB1* and *KRAS* (Supplementary Table 3), while previous findings describe that these specific co-mutations can be associated with wore response to ICI therapy^18^. In this case, the molecular profile did not point to mechanisms of resistance but is a major reduction of total CD8^+^ T cells and upregulation of Tim-3 in the resistant sample might explain the progression after the initial response. Due to clinical implications, the site of tissue sampling might vary. Very often the primary tumor cannot be biopsied and a distant metastasis is analyzed. However, different lesions might vary in their genomic background^109^. Nevertheless, driver mutations are often conserved across different metastases and primary tumors and metastases share often their genomic background^110^ while in general different metastases from the same patient have up to a 50 – 60% genomic overlap of the analyzed genes and a rather large heterogeneity in different immune cell infiltrates^111^.

Despite the small number of cases analyzed, we have identified events intrinsic and extrinsic to cancer cells occurring at resistance. This information derived from IMC analysis integrated with genomic data can generate new hypothesis to be explored in preclinical studies with possible clinical implications for treatment decisions. These findings moreover support the importance of reanalyzing tumors at times of resistance to develop personalized treatments for patients with NSCLC.

## Methods

### Patients and response assessment

Clinical data and tumor material from seven patients with stage IV NSCLC undergoing anti-PD-1 immunotherapy at the University Hospital Zurich, Switzerland between 2015 and 2019 were included in this study. Response assessment was performed according to the Immune Response Evaluation Criteria in Solid Tumors (iRECIST) criteria^112^. Tumor tissue was collected from patients who initially responded to immunotherapy (tumor sample before start of immunotherapy treatment: Response) and at resistance to immunotherapy (Resistance). The study was approved by the Cantonal Ethical Committee Zurich (KEK-ZH-2018-01919, KEK-ZH-2020-02566). All patients included in the study provided informed written consent. The study was performed in accordance with the declaration of Helsinki.

The sex of the patient is reported in Supplementary Fig. 3, but was not included in the further analysis, and clinical data are reported according to the STROBE guidelines.

### Tumor sample preparation for sequencing

The patient material was formalin-fixed and paraffin-embedded (FFPE). A 3-µm section was stained with hematoxylin/eosin, and the tumor area as well as regions of normal tissue were marked by an experienced pathologist. Punch biopsies of 0.4 mm in diameter were taken from both areas for DNA/RNA isolation.

### DNA/RNA isolation

DNA and RNA of normal and tumor tissue were isolated using the Maxwell® 16 FFPE Tissue Plus LEV DNA Purification Kit (Promega) or the Maxwell® 16 LEV RNA FFPE Purification Kit (Promega), respectively, according to the manufacturer’s instructions. Both DNA and RNA were quantified by a fluorometric assay (Qubit, Thermo Fisher Scientific).

### Foundation one testing

Tumor DNA was extracted from FFPE tissues and analyzed using the FoundationOneCDx™ (FOneCDx) assay. The FOneCDx assay detects genomic alterations in a panel of 324 genes. In addition, the genomic signatures tumor mutational burden and microsatellite instability are reported. For sequencing, the Illumina® HiSeq 2500 platform was used. Hybrid capture-selected libraries were sequenced (targeting >500x median coverage with >99% of exons at coverage >100x). Sequence data were analyzed by a customized analysis pipeline designed to detect all classes of genomic alterations, including base substitutions, indels, selected genomic rearrangements (e.g., gene fusions), and copy number alterations (amplifications and homozygous gene deletions). The threshold used in FoundationOneCDx for identifying a copy number amplification was 4 for *ERBB2* and 6 for all other genes (FMI technical information sheet).

### Oncomine focus assay

The Oncomine Focus Assay panel interrogates 52 genes for the presence of mutations, small insertion/deletions, copy number alterations, and fusions. Since DNA was used as input material in our study, the fusion part of the assay was not performed. In brief, DNA was isolated using Maxwell 16 FFPE Plus LEV DNA Purification Kit (Promega). The DNA concentration was measured with Qubit, and 10 ng was used for library preparation. Emulsion-PCR, enrichment, and chip loading were carried out on the Ion Chef with the Ion 510 & Ion 520 & Ion 530 Kit or 540 Kit. The S5 platform was used for sequencing with the Ion S5 Sequencing Kit (Life Technologies/Thermo Fisher Scientific). Alignment, variant calling, and annotation were performed with the Ion Reporter Software 5.10 workflow from Thermo Fisher Scientific (Oncomine Focus w2.4 – DNA - Single Sample; Filter chain: Oncomine 5% CI, CNV ploidy >= gain of 2 over normal).

### Whole exome sequencing

DNA integrity number was estimated using a Genomic DNA Screen Tape Assay (Agilent). The PReCR mix (NEB) was used after fragmentation of the DNA (Covaris E220: Duty cycle = 10%, Peak Intensity = 175, Cycles = 200, Time = 240 s) to overcome amplification bias due to DNA damage during the library preparation and to improve the single nucleotide polymorphism calling efficiency. After bead clean up (AMPure beads, Beckman Coulter) Illumina’s TruSeq DNA Nano library kit was used with slight modifications: no size selection, 13 cycles of PCR. The samples were enriched for the whole exome with Agilent’s Sure Select Target enrichment regents (V6 + UTR). To block the complete P5/P7 adapter structure of the library molecules, the IDT blocking reagent for TruSeq DNA Nano libraries replaced the blocking oligos of the Agilent protocol. Accordingly, the PCR primers used during post-capture PCR were the TruSeq DNA Nano Primer Cocktail Mix (Illumina), and the annealing temperature was set to 60 °C. The exome-enriched libraries were sequenced on Illumina’s HiSeq 4000 in 75-bp paired-end mode.

### Whole genome sequencing

The libraries for whole genome sequencing were processed as described in the whole exome workflow, but fragmentation time was set to 120 s and the libraries were amplified with 8 cycles of PCR. Sequencing was performed on Illumina’s HiSeq 4000 in 75-bp paired-end mode.

### Whole exome and whole genome sequencing data analysis

Raw whole exome sequencing (WES) and whole genome sequencing (WGS) data were processed as follows: SeqPurge^113^ was used to trim adapters. The trimmed reads were mapped to the human reference genome hg19 using BWA^114^. WGS and WES mappings were post-processed using Picard MarkDuplicates. WES mappings were further processed using GATK (v3.5) IndelRealigner and BaseRecalibrator as well as BamClipOverlap from the ngs-bits library^115^.

Somatic variant calling was performed on WES tumor-normal pairs. SNVs were called using three somatic variant callers: MuTect^116^, VarScan2^117^, and Strelka^118^. Small somatic InDels were called using VarScan2 and Strelka. In order to identify variants with greater confidence and reduce the number of false positive calls only SNVs and InDels reported by at least two callers were considered in the subsequent analyzes. Default parameters of the callers were used with the following exceptions: For MuTect a BAQ gap open penalty of 30 was used. For VarScan2 a minimum coverage threshold of 10 was employed. In addition, a minimum read support of two and a minimum variant frequency of 0.01 was adopted. The adjusted P-value threshold for somatic variants was set to 0.01 and the minimum variant frequency in normal to report a loss of heterozygosity was set to 0.25. The pipeline from reads to unannotated WES variant calls is based on the framework described in ref. ^119^.

Copy number variants (CNVs) were called using BIC-seq2^120^ on WGS tumor-normal pairs. Only CNVs called with a *p*-value smaller than or equal to 0.05 were considered in the subsequent analysis.

### RNA sequencing

RNA was isolated from FFPE tissue and quantified using the TapeStation RNA standard sensitivity kit (Agilent). Due to considerable RNA degradation, the SMARTer® Stranded Total RNA Seq Kit v2 (Takara) was used to prepare cDNA by universal priming (without fragmentation) and to deplete ribosomal cDNA with ZapR v2 and R Probes v2. The libraries were quantified by Tapestation D1000 (Agilent) measurements, and fragment sizes between 290 – 407 bp were detected. The libraries were sequenced on a HiSeq 4000 platform using 75 cycles paired end targeting ~50 M reads per sample.

### RNA sequencing data analysis

RNA-seq data (Illumina HiSeq4000, 2x76bp) was processed using the International Cancer Genome Consortium (ICGC) pipeline: Reads were aligned to the human reference hg19 using STAR (version 2.4.3a) and gene counts were determined using HTSeq (version 0.9.1). Gene set variation analysis was performed using the R package GSVA^121^ and a set of 12 gene sets from the MSigDB Hallmark Gene Sets. The resulting gene set enrichment scores per gene set and sample were displayed as heatmaps. The R package DESeq2^122^ was used to determine log2 fold changes (log2FC) in gene expression between tumor pairs. Genes were ranked based on the log2FC, and the 100 genes showing the strongest decrease in expression from response to resistance (negative log2FC) were selected for further analysis.

Over- and underexpression of genes was determined by comparison of the sample gene expression to gene expression in the TCGA LUAD cohort^123^. TCGA LUAD gene expression data was retrieved from the Broad GDAC Firehose^124^. Based on the TCGA-RNAseqv2 pipeline RSEM was used to quantify transcripts for the individual samples. Quantile normalized gene counts were then compared to the gene expression distribution of the TCGA cohort. With Q1 being the 25th percentile and Q3 being the 75th, we consider expression more than 1.5*IQRs (interquartile range; Q3-Q1) below Q1 of the TCGA cohort underexpression. Expression of more than 1.5*IQRs above Q3 is considered overexpression.

### Tissue preparation for IMC

To enable imaging mass cytometry (IMC) imaging, all tissue sections were stained with an IMC panel of metal-tagged antibodies (Supplementary Table 7). Here, the tissue sections were first deparaffinized and rehydrated using HistoClear and a graded ethanol series. The rehydrated tissues were then incubated in a decloaking chamber for 30 min at 95 °C in HIER buffer (pH 9.2) before blocking in buffer containing 3% BSA. The tissues were stained with the metal-tagged anti-CD3 and anti-pan Cytokeratin antibodies from the panel at 4 °C overnight. On the following day, the respective secondary fluorescence-labeled antibodies (Alexa fluor 555 and Alexa fluor 750) were incubated for an hour at room temperature before staining with Hoechst. Whole slide scans were performed before incubating again overnight with the remaining metal-labeled antibodies of the panel. Finally, the slides were stained with an iridium intercalator before being dried and measured using IMC.

### IMC imaging

For imaging, regions were selected based on the pan Cytokeratin and CD3 immunohistochemistry fluorescence staining. On the same tumor, one CD3 high area and a CD3 low area were chosen (Suppl Fig. 1). To detect the selected areas in IMC, brightfield scans were done using the CyTOF software before imaging was carried out using Hyperion Imaging System coupled to a Helios time-of-flight mass cytometer. For every patient, four representative areas (800 µm^2^) were ablated at a laser frequency of 400 Hz and at a nominal resolution of 1 µm. To account for machine performance and spillover between channels, the machine was calibrated on a daily basis and a spillover slide containing all metal tags was measured^36^.

### IMC analysis

Tiff files were generated from the IMC raw data using the Bodenmiller lab’s IMC segmentation pipeline (github.com/BodenmillerGroup/ImcSegmentationPipeline [github.com/BodenmillerGroup/ImcSegmentationPipeline]), customized python script before segmenting cells using Ilastik pixel classification (v1.3.3) based on nuclear and membrane staining. Cell masks were created based on the ilastik (probability maps using CellProfiler (v3.1.9). Mean intensities for every marker were calculated for every cell. The raw counts were arcsinh transformed using cofactor 1. The single-cell data was ultimately analyzed using R (v3.6). Clustering was performed by building a SNNgraph (scran: v1.14.6) jaccard) and Louvain clustering (igraph: v1.2.5).

### Immunohistochemistry

FFPE tumor samples were cut into 2-µm sections, mounted on glass slides, and dried overnight. The next day tumor sections were deparaffinized, rehydrated, and stained with the following antibodies: CD3 (clone LN10), PDL1 (clone E1L3N), CD8 (clone C8/144B), TIGIT (clone TG1). A detailed description of the antibodies, dilutions, and conditions are listed in Supplementary Table 8. The immunohistochemical staining was performed using Ventana BenchMark Ultra (Roche) as recently described^125^. The entire available tumor slides were analyzed using the qualitative pathology and bioimaging analysis software QuPath version 0.3.2^126^ to define a classifier that recognizes positive staining automatically.

### Statistics and reproducibility

No statistical method was used to predetermine the sample size. No data were excluded from the analyzes.

### Reporting summary

Further information on research design is available in the Nature Portfolio Reporting Summary linked to this article.

### Supplementary information

Supplementary Information

Description of Additional Supplementary Files

Supplementary Data 1

Supplementary Data 2

Reporting Summary

### Source data

Source Data

## Data Availability

The IMC data generated in this study have been deposited in the Zenodo database under the link https://zenodo.org/deposit/8041882 (https://zenodo.org/record/8041882). The raw sequencing data are protected and are not available due to data privacy laws, access can be obtained upon request. The processed sequencing data generated in this study are provided in the Supplementary Information/Source Data file. Following database were used for analysis. **GSVA MSigDB**: Link: https://data.broadinstitute.org/gsea-msigdb/msigdb/release/6.2/h.all.v6.2.symbols.gmt, Version: GSEA MSigDB Release 6.2. **WES& WGS**: Human reference ucsc.hg19.fasta: Link: gs://gatk-legacy-bundles, Version: Gatk bundle 2.8. **RNASeq**: Human reference fasta, gff3 and gtf: Link: https://www.gencodegenes.org/human/release_19.html, Version: release 19. **Cohort comparison**: TCGA_LUAD_rnaseqV2_RSEM_genes_normalized, Link: https://gdac.broadinstitute.org/, Version: V2 Source data are provided with this paper.

## Code availability

The code used for the IMC analysis is available on Zenodo https://zenodo.org/deposit/8041882. For genomic analysis the code and software versions used are available on Github https://github.com/Lab-Curioni/Acquired_Resistance_ICI_NSCLC.
