# Supplementary material for: Acquired resistance to anti-PD1 therapy in patients with NSCLC associates with immunosuppressive T cell phenotype
Source: Nat Commun. 2023 Aug 24;14:5154. doi: 10.1038/s41467-023-40745-5 (PMC10449840; doi:10.1038/s41467-023-40745-5)
Supplement: Supplementary file 1 — Supplementary Information [file 41467_2023_40745_MOESM1_ESM.pdf]

Supplemental information for the manuscript:

Acquired resistance to anti-PD1 therapy in patients with NSCLC associates with immunosuppressive T cell phenotype

Stefanie Hiltbrunner[1,2,3,4], Lena Cords[4,5,6,7], Sabrina Kasser[2,4], Sandra N. Freiberger[8], Susanne Kreutzer[9], Nora C. Toussaint[10,11], Linda Grob[10,11], Isabelle Opitz[12], Michael Messerli[3,13], Martin Zoche[8], Alex Soltermann[8], Markus Rechsteiner[8], Maries van den Broek[4,14], Bernd Bodenmiller[4,5,6], and Alessandra Curioni-Fontecedro[1,2,3,4,15*]

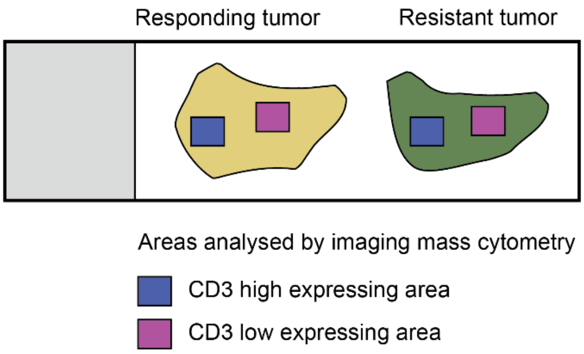

Supplementary Fig. 1: Schematic overview on the analyzed areas by IMC. In the responding and resistant tumors a CD3 high and a CD3 low area were selected.

a **b**

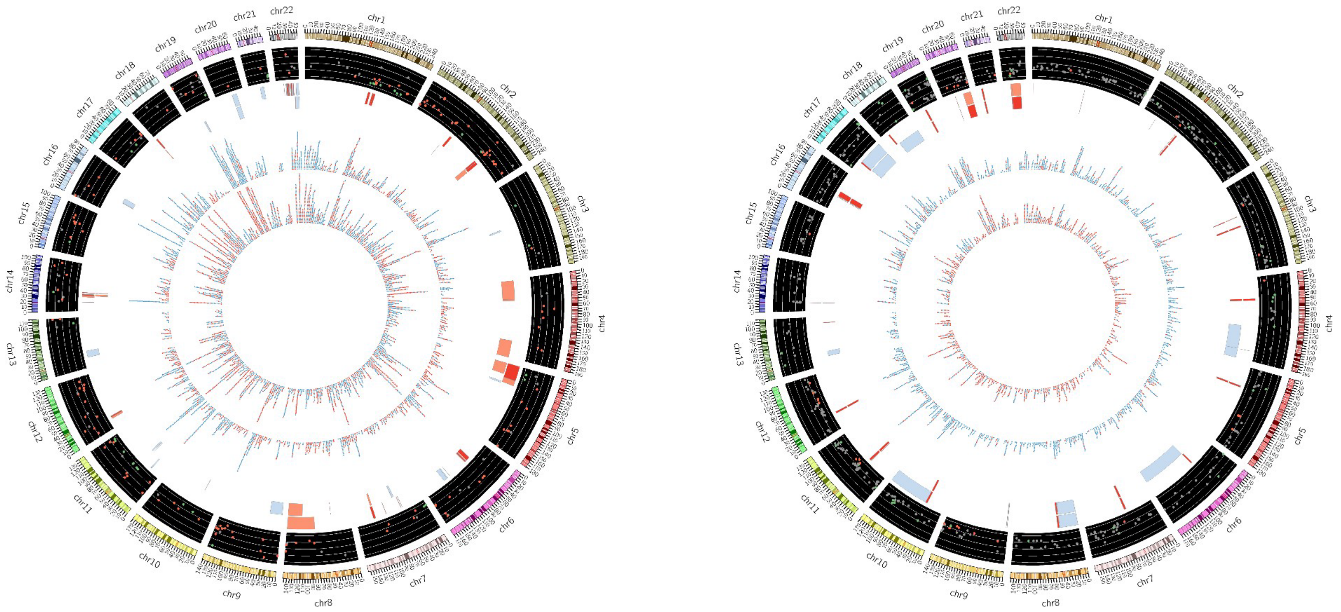

Supplementary Fig. 2: Overview on sequencing data from (a) patient 1, (b) patient 7. The black outer ring indicates all SNVs plotted according to their frequency, green dots mutations only at response, red dots mutations only at resistance. The ring in the middle indicates all CNVs, inner ring at response, outer ring at resistance, dark red: copy number > 4, light red: copy number > 2, light blue: copy number < 2, dark blue: copy number = 0, the two rings in the center represent RNA sequencing data, red: overexpression, blue: underexpression, inner ring at response, outer ring at resistance, data related to the TCGA cohort.

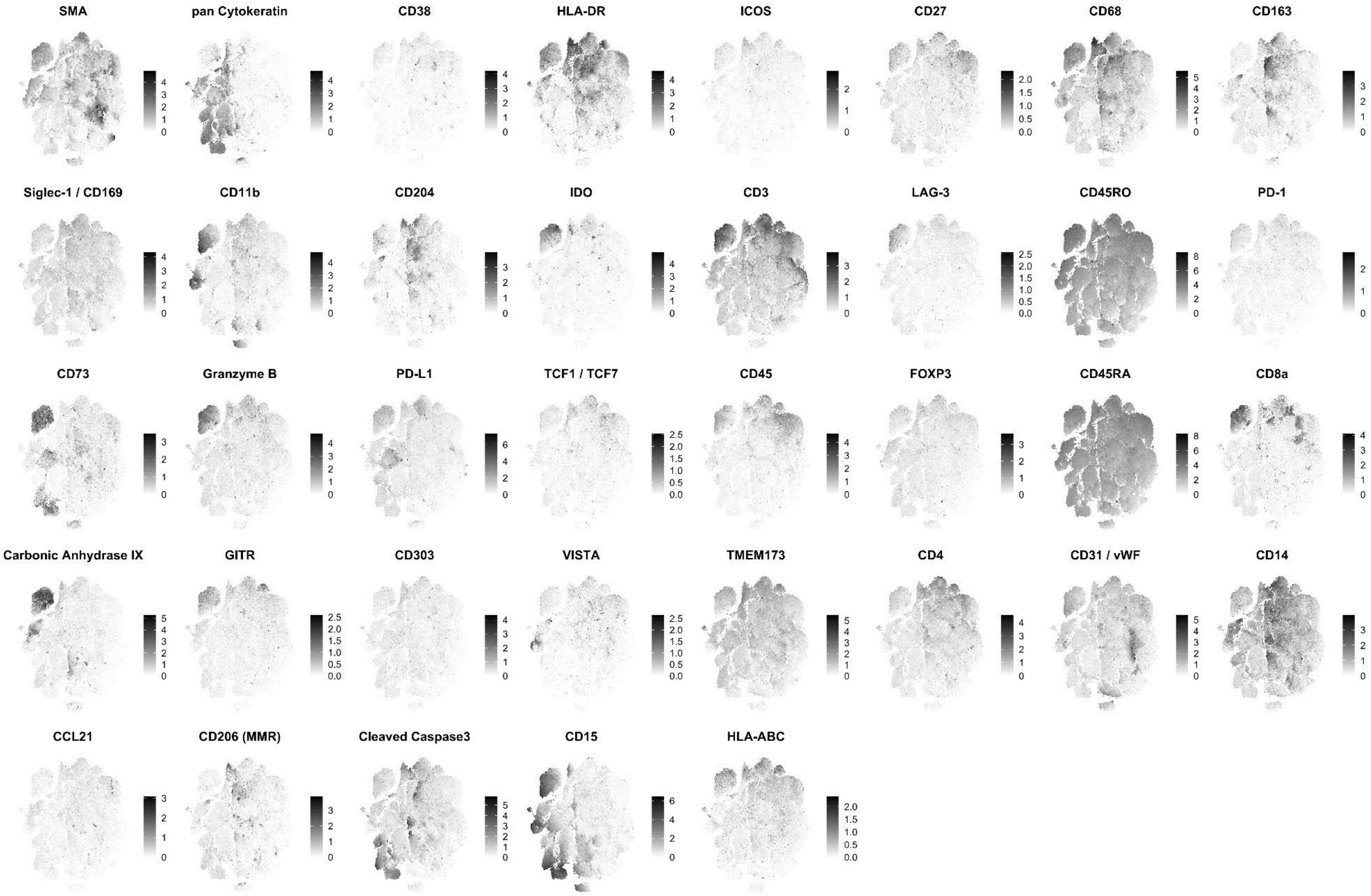

Supplementary Fig. 3: t-SNE plots over all cell markers used in the study

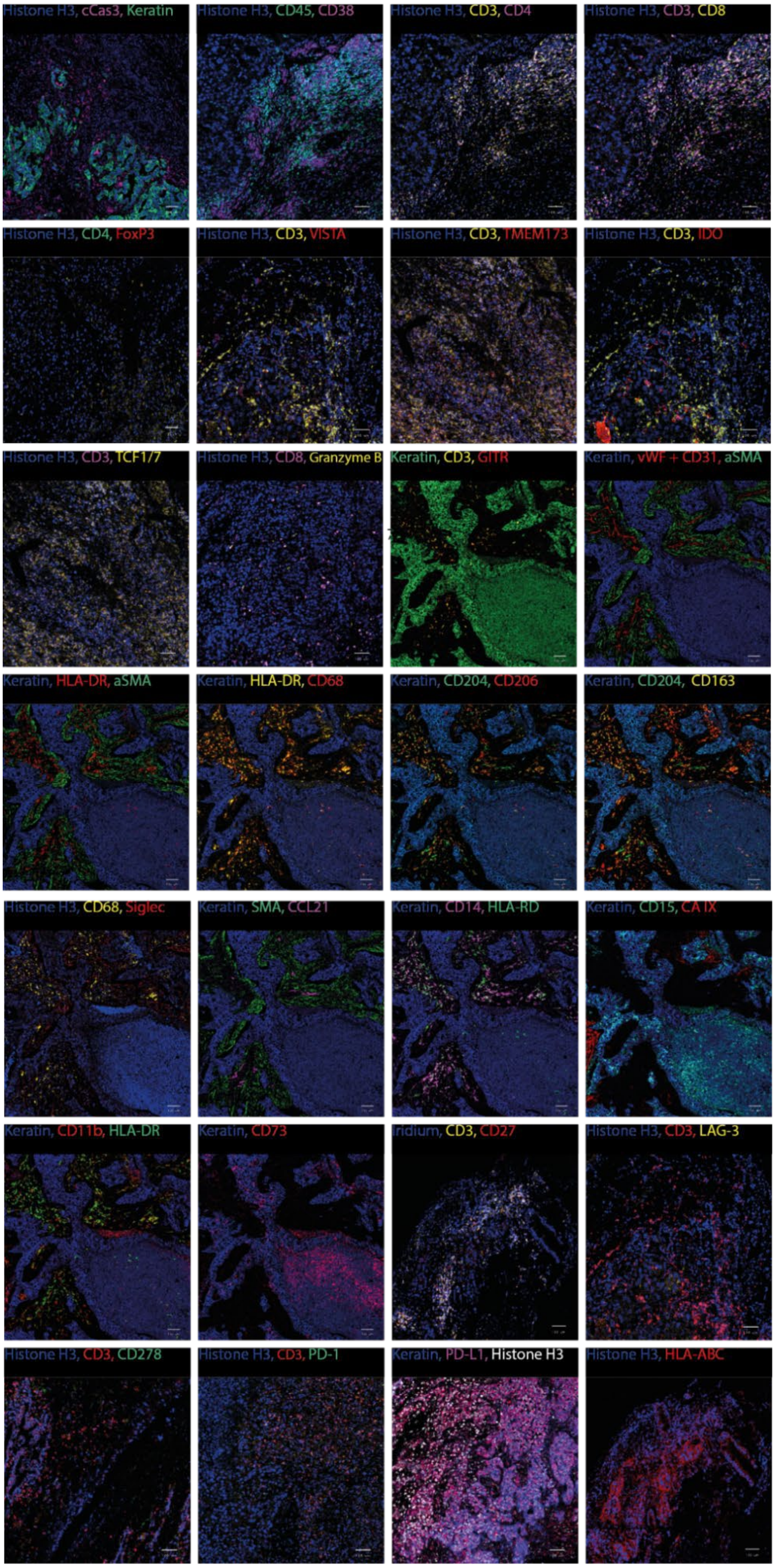

Supplementary Fig. 4: Representative immunofluorescence stainings of all IMC antibodies used in the study.

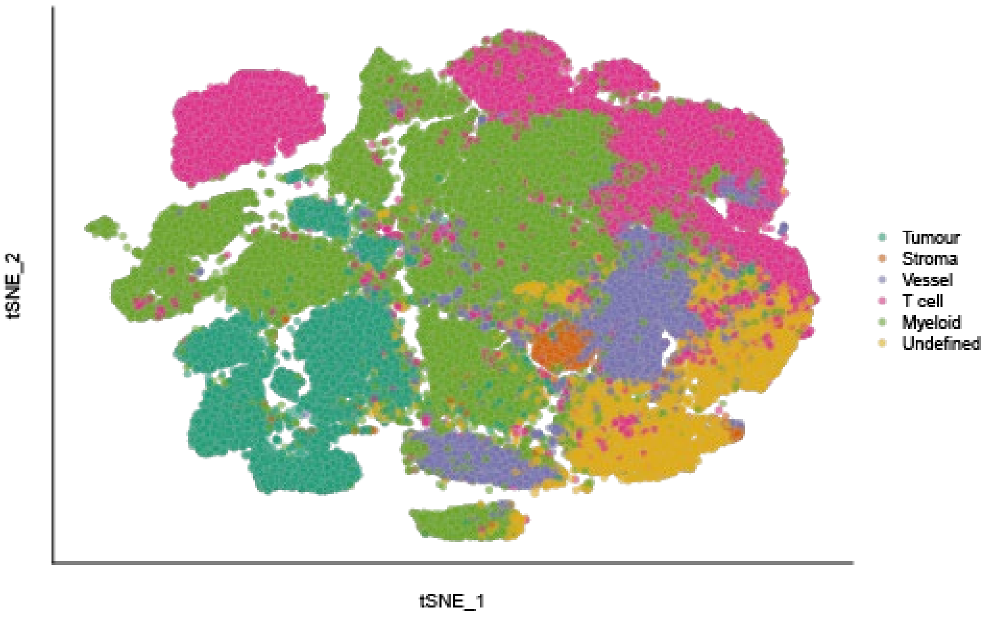

Supplementary Fig. 5: t-SNE plot colored-coded to the cell subtypes depicts clear separation between the different subtypes, related to Fig. 4 and 5

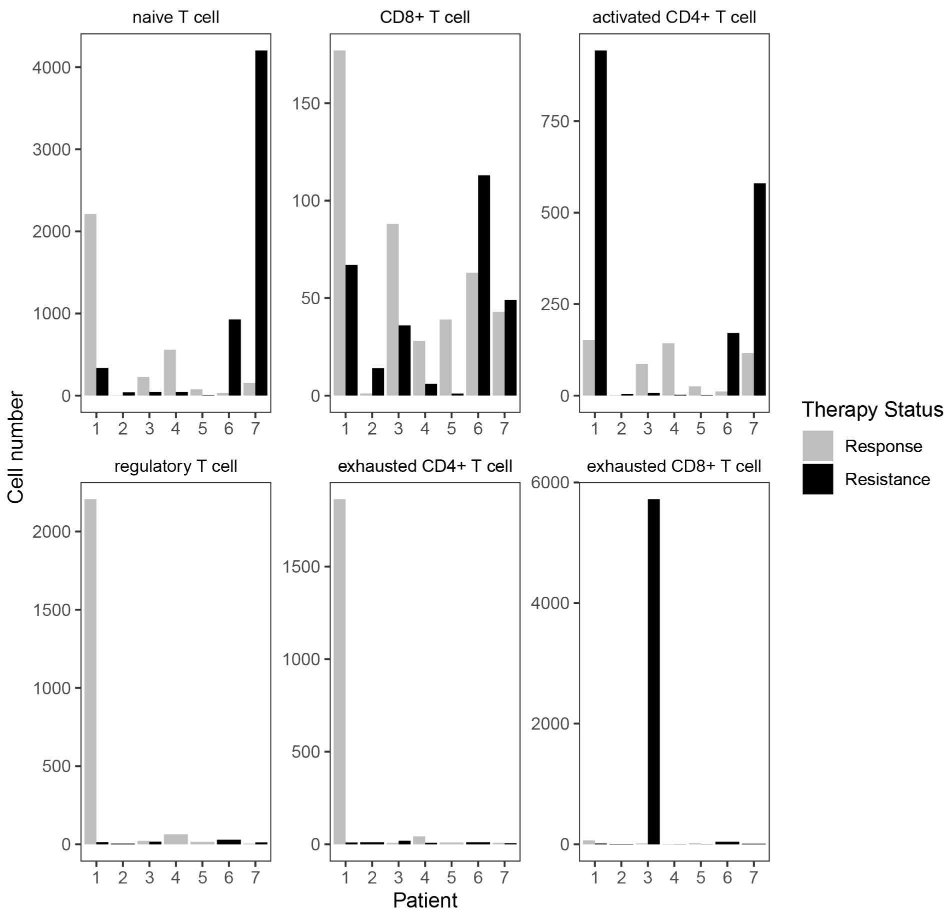

Supplementary Fig. 6: Numbers of T cell subsets at response and resistance, related to Fig. 3

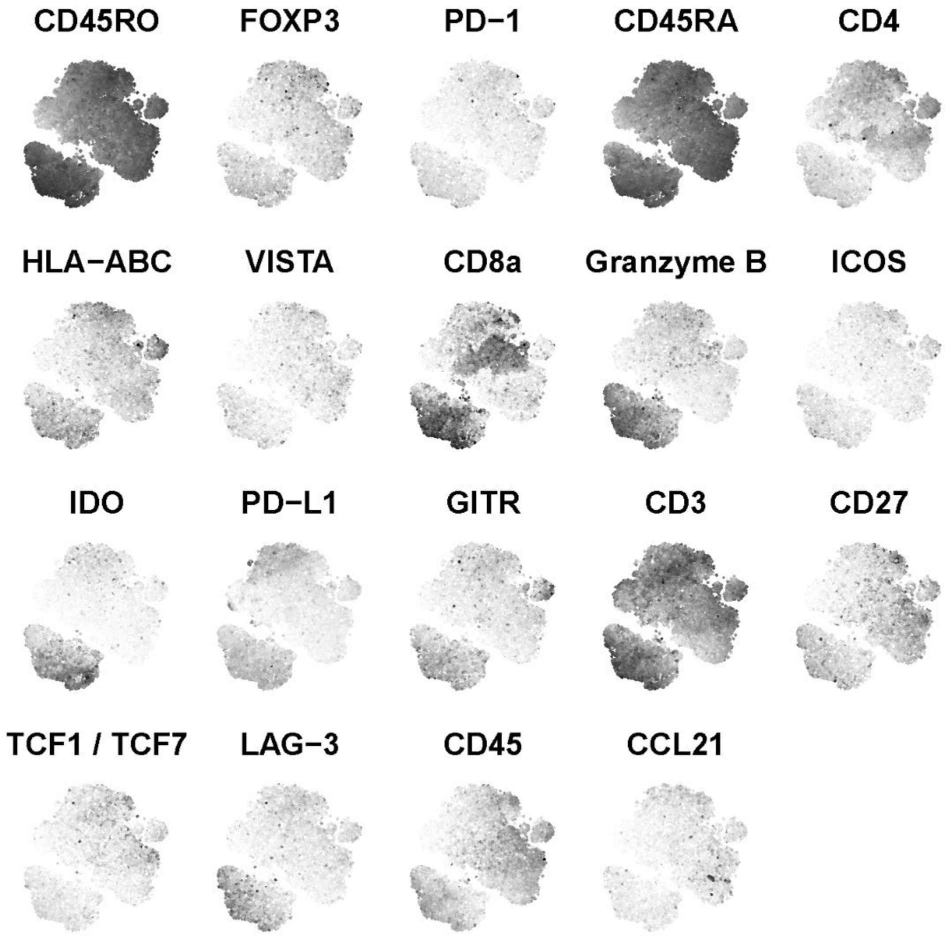

Supplementary Fig. 7: t-SNE plots over all T cell markers, related to Fig. 4

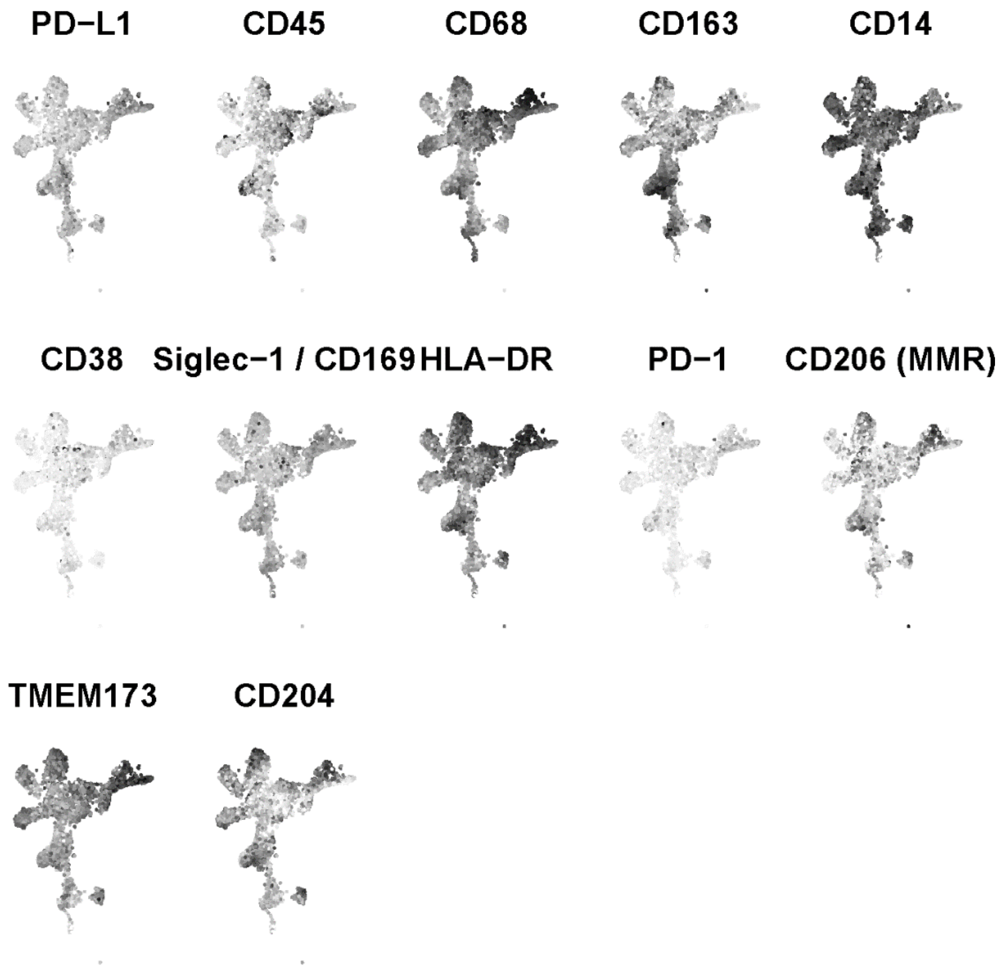

Supplementary Fig. 8: t-SNE plots over all macrophage cell markers, related to Fig. 5

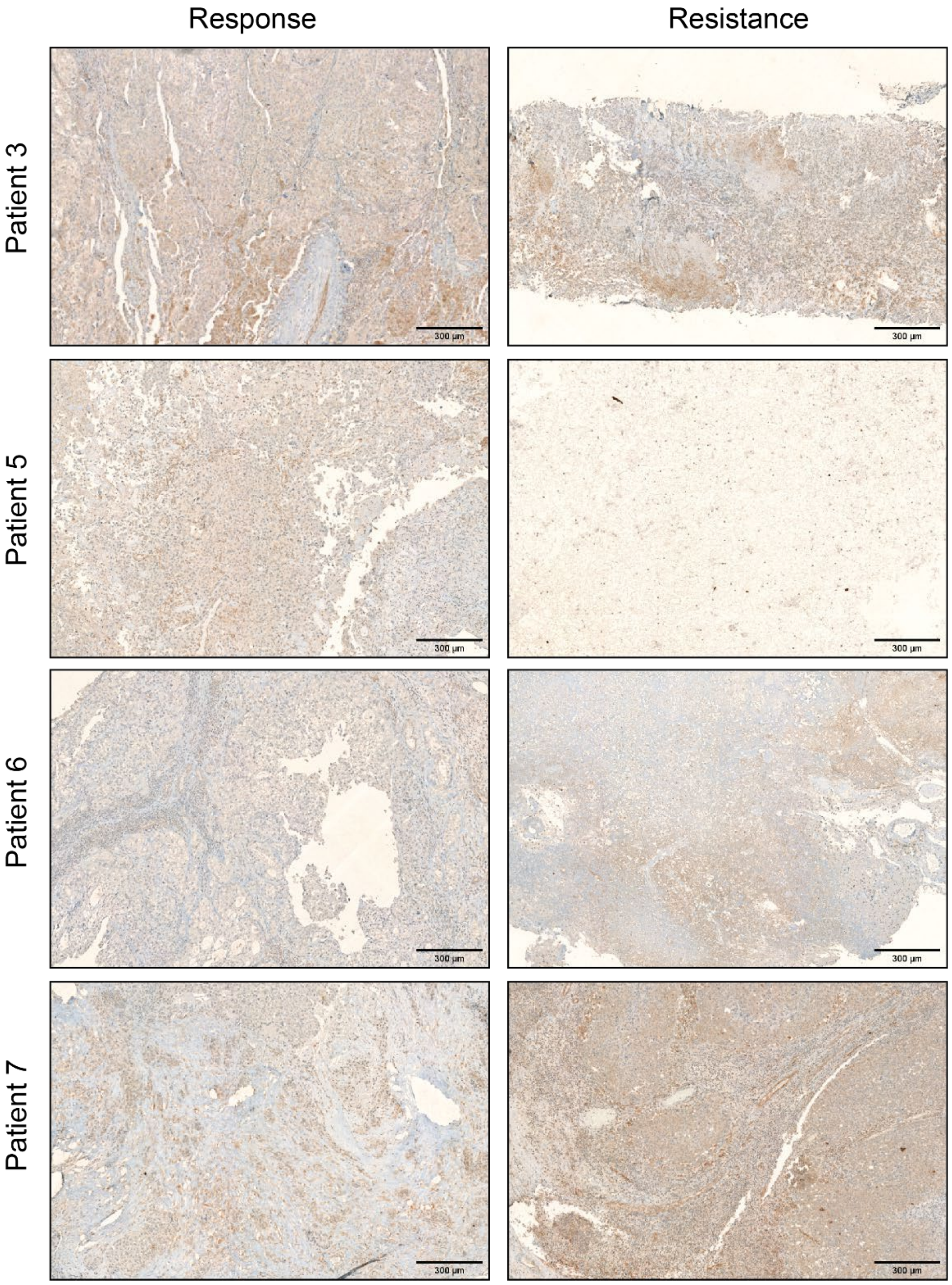

Supplementary Fig.9: IHC of Tim-3 at response and at resistance in patients 3, 5, 6 and 7, magnification 4x, scale bar 300 µm, one tumor per patient for each time point was analyzed.

Supplementary Table 1. Patient history

	Stage at diagnosis and histology	Treatment lines	Time of resistance after start of CPI, months (m)
**Patient 1**	IIIB (Ad)	CRT → anti-PD1	10m
**Patient 2**	IV (Ad)	CRT → anti-PD1+anti-CTLA4	6m
**Patient 3**	IV (Ad)	TKI → anti-PD1	7m
**Patient 4**	IV (Ad-Sq)	CRT → Ch → anti-PD1	9m
**Patient 5**	IV (Ad)	Anti-PD1	8m
**Patient 6**	IV (Ad)	CRT → anti-PD1	8m
**Patient 7**	IV (Ad)	Ch → anti-PD1	15m

Ad: Adenocarcinoma; Ad-Sq: Adenosquamous; CRT: Chemoradiotherapy; Ch= Chemotherapy; CPI: checkpoint inhibitors

Supplementary Table 2. Genes with short variants of patient 7 only present at resistance

<table>
<tr><th>Chromosome</th><th>Position</th><th>Change</th><th>Frequency</th><th>Genes</th><th>Annotation</th></tr>
<tr><td>chr1</td><td>82409203</td><td>G>T</td><td>34.2</td><td>ADGRL2</td><td>ADGRL2:c.948G>T|p.Met316Ile</td></tr>
<tr><td>chr2</td><td>67631048</td><td>G>T</td><td>31.6</td><td>ETAA1</td><td>ETAA1:c.1234G>T|p.Asp412Tyr</td></tr>
<tr><td>chr9</td><td>18775811</td><td>T>C</td><td>29.6</td><td>ADAMTSL1</td><td>ADAMTSL1:c.2468T>C|p.Leu823Pro</td></tr>
<tr><td>chr9</td><td>126214571</td><td>T>A</td><td>26.3</td><td>DENND1A</td><td>DENND1A:c.1283A>T|p.Lys428Met</td></tr>
<tr><td>chr19</td><td>7523450</td><td>C>G</td><td>23.1</td><td>ARHGEF18</td><td>ARHGEF18:c.1670C>G|p.Ala557Gly;ARHGEF18:c.1196C>G|p.Ala399Gly</td></tr>
<tr><td>chr12</td><td>97051883</td><td>A>T</td><td>22.2</td><td>CFAP54</td><td>CFAP54:c.5324A>T|p.His1775Leu</td></tr>
<tr><td>chr12</td><td>120106195</td><td>C>T</td><td>21</td><td>PRKAB1</td><td>PRKAB1:c.146C>T|p.Ser49Phe;PRKAB1:c.146C>T|</td></tr>
<tr><td>chr5</td><td>108207909</td><td>G>T</td><td>20</td><td>FER</td><td>FER:c.919G>T|p.Val307Leu;FER:c.394G>T|p.Val132Leu</td></tr>
<tr><td>chr5</td><td>140803085</td><td>C>G</td><td>19.3</td><td>PCDHGA11;PCDHGB8P;PCDHGB7;PCDHGA1;PCDHGA2;PCDHGA3;PCDHGB1;PCDHGA4;PCDHGB2;PCDHGA5;PCDHGB3;PCDHGA6;PCDHGA7; PCDHGB4;PCDHGA8;PCDHGB5;PCDHGA9;PCDHGB6;PCDHGA10</td><td>PCDHGA11:c.2291C>G|p.Ser764Trp;PCDHGB8P:n.-2768C>G|;PCDHGB7:c.*3232C>G|;PCDHGA1:c.2422-71289C>G|;PCDHGA2:c.2425-71289C>G|;PCDHGA3:c.2425-71289C>G|;PCDHGB1:c.2409+70849C>G|;PCDHGA4:c.2514+65897C>G|;PCDHGB2:c.2421+60962C>G|;PCDHGA5:c.2421+56767C>G|;PCDHGB3:c.2415+50709C>G|;PCDHGA6:c.2424+47011C>G|;PCDHGA7:c.2424+38195C>G|;PCDHGB4:c.2397+33237C>G|; PCDHGA8:c.2424+28281C>G|;PCDHGB5:c.2397+22994C>G|;PCDHGA9:c.2424+18142C>G|;PCDHGB6:c.2418+12898C>G|;PCDHGA10:c.2436+7907C>G|;PCDHGB7:c.2415+3244C>G|;PCDHGA11:c.1878+413C>G|</td></tr>
<tr><td>chr9</td><td>71679882</td><td>G>T</td><td>17.9</td><td>FXN</td><td>FXN:c.413G>T|p.Gly138Val;FXN:c.413G>T|</td></tr>
<tr><td>chr9</td><td>140481452</td><td>C>T</td><td>17.9</td><td>ZMYND19</td><td>ZMYND19:c.326G>A|p.Arg109Gln</td></tr>
<tr><td>chr7</td><td>73119495</td><td>G>A</td><td>17.4</td><td>STX1A</td><td>STX1A:c.268C>T|p.Arg90Cys</td></tr>
<tr><td>chr20</td><td>16021914</td><td>G>T</td><td>16.7</td><td>MACROD2</td><td>MACROD2:c.1222G>T|p.Gly408Cys;MACROD2:c.517G>T|p.Gly173Cys</td></tr>
<tr><td>chr15</td><td>49074401</td><td>G>A</td><td>16.5</td><td>CEP152</td><td>CEP152:c.1348C>T|p.Gln450*</td></tr>
<tr><td>chr12</td><td>72029263</td><td>C>A</td><td>16.4</td><td>ZFC3H1</td><td>ZFC3H1:c.2198G>T|p.Gly733Val</td></tr>
<tr><td>chr21</td><td>46078009</td><td>T>A</td><td>16</td><td>KRTAP12-3;KRTAP12-4;TSPEAR</td><td>KRTAP12-3:c.113T>A|p.Val38Glu;KRTAP12-4:c.-3478A>T|;TSPEAR:c.82+53339A>T|;TSPEAR:c.-123+32451A>T|</td></tr>
<tr><td>chr12</td><td>78400279</td><td>T>C</td><td>15.5</td><td>NAV3</td><td>NAV3:c.961T>C|p.Ser321Pro</td></tr>
<tr><td>chr3</td><td>51378776</td><td>G>C</td><td>15.4</td><td>DOCK3</td><td>DOCK3:c.3875G>C|p.Gly1292Ala</td></tr>
<tr><td>chr12</td><td>81283136</td><td>G>A</td><td>14.9</td><td>LIN7A</td><td>LIN7A:c.95C>T|p.Ala32Val;LIN7A:c.-12-41035C>T|;LIN7A:n.276-41035C>T|;LIN7A:n.288C>T|</td></tr>
<tr><td>chr2</td><td>84936633</td><td>G>T</td><td>14.3</td><td>DNAH6</td><td>DNAH6:c.9215G>T|p.Gly3072Val</td></tr>
<tr><td>chr4</td><td>9785030</td><td>C>A</td><td>13.8</td><td>DRD5</td><td>DRD5:c.1377C>A|p.Cys459*</td></tr>
<tr><td>chr9</td><td>107289445</td><td>C>T</td><td>13.8</td><td>OR13C4</td><td>OR13C4:c.46G>A|p.Gly16Arg</td></tr>
<tr><td>chr22</td><td>17670893</td><td>G>T</td><td>13.6</td><td>CECR1</td><td>CECR1:c.911C>A|p.Ser304Tyr;CECR1:c.188C>A|p.Ser63Tyr;CECR1:c.785C>A|p.Ser262Tyr;CECR1:c.551C>A|p.Ser184Tyr</td></tr>
<tr><td>chr11</td><td>74632266</td><td>C>T</td><td>13.3</td><td>XRRA1</td><td>XRRA1:c.625G>A|p.Ala209Thr;XRRA1:c.-75G>A|</td></tr>
<tr><td>chr5</td><td>108673054</td><td>C>T</td><td>12.5</td><td>PJA2</td><td>PJA2:c.2005G>A|p.Gly669Arg</td></tr>
<tr><td>chr20</td><td>60902010</td><td>C>T</td><td>12.3</td><td>LAMA5</td><td>LAMA5:c.5125G>A|p.Gly1709Ser</td></tr>
<tr><td>chr11</td><td>62295656</td><td>A>T</td><td>12.2</td><td>AHNAK</td><td>AHNAK:c.6233T>A|p.Val2078Asp;AHNAK:c.342+6819T>A|</td></tr>
</table>

chr20	31815368	C>A	11.6	BPIFA3	BPIFA3:c.710C>A|p.Thr237Asn;BPIFA3:c.602C>A|p.Thr201Asn
chr20	40113115	G>A	11	CHD6	CHD6:c.2134C>T|p.Arg712Cys
chr9	112141911	G>T	10.8	PTPN3	PTPN3:c.2667C>A|p.Ser889Arg;PTPN3:c.1806C>A|p.Ser602Arg;PTPN3:c.1671C>A|p.Ser557Arg;PTPN3:c.2274C>A|p.Ser758Arg;PTPN3:c.2139C>A|p.Ser713Arg;PTPN3:c.2532C>A|p.Ser844Arg;PTPN3:n.1549C>A|
chr3	164906102	G>T	10.7	SLITRK3	SLITRK3:c.2517C>A|p.His839Gln
chr11	69063836	C>A	5.4	MYEOV	MYEOV:c.919C>A|p.Leu307Ile;MYEOV:c.745C>A|p.Leu249Ile

Supplementary Table 3. Genomic characteristics of all patients included in the study (Foundation One analysis)

Patient	Sex	Histology	Time point	Status	Specimen	Short Variants	Copy number alteration	Rearrangements	TMB-Status
1	male	AC	Response	na	Lung				
			Resistance	Completed	Soft Tissue	*ARID1A* , *ATR, CTNNB1, KRAS G12C, MUTYH* splice site, *TP53*	*GNAS, KRAS G12C, MCL1, MET, NFKBIA, NKX2-1, RAD21, VEGFA*		13 Muts/Mb
2	male	AC	Response	na	Liver				
			Resistance	Completed	Soft Tissue	*DNMT3A, KRAS G12V, NFKBIA*		*CDKN2A-CDKN2A* fusion	8 Muts/Mb
3	male	AC	Response	Completed	Lung	*KRAS G13C, NF1* splice site, *NF1, SMARCA4, TP53, U2AF1*			34 Muts/Mb
			Resistance	na	Lung				
4	female	SCC	Response	Completed	Lung	*KRAS G13D, NFE2L2, PIK3CB, TP53* splice site	*RICTOR*		4 Muts/Mb
			Resistance	na	Lung				
5	male	AC	Response	Completed	Soft Tissue	*MAP2K1, TP53*	*NFKBIA*		19 Muts/Mb
			Resistance	Completed	Lymph Node	*MAP2K1, SMARCA4, TP53*			14 Muts/Mb
6	female	AC	Response	Completed	Brain	*KRAS G13C, NOTCH1, STK11, TP53*	*MCL1, RPTOR*		11 Muts/Mb
			Resistance	na	Brain				
7	male	AC	Response	Completed	Pleura	*CREBBP, FANCA, TP53*			8 Muts/Mb
			Resistance	Completed	Lymph Node	*CREBBP, FANCA, TP53*	*ARFRP1, AURKA, CRKL, GNAS, MAPK1, ZNF217*		6 Muts/Mb

AC: Adenocarcinoma, SSC: Squamous cell carcinoma

Supplementary Table 4. Genes with copy number alterations (from WGS) in sample from tumor from patient #7 isolated at resistance

Gene	Chromosome	Start	End	Copy Number	Call Type	p value	log2 ratio
JB175072	chr16	33964385	34196972	6.4	2	1.78E-05	1.67401
LOC646813	chr11	50323722	55026423	4.7	2	0.00014859	1.23908
TRNA_Lys	chr11	50323722	55026423	4.7	2	0.00014859	1.23908
OR4A5	chr11	50323722	55026423	4.7	2	0.00014859	1.23908
OR4C46	chr11	50323722	55026423	4.7	2	0.00014859	1.23908
DQ579288	chr4	49093177	52661966	4.1	2	0.00205358	1.03634
DQ583161	chr4	49093177	52661966	4.1	2	0.00205358	1.03634
DQ579969	chr4	49093177	52661966	4.1	2	0.00205358	1.03634
DQ593719	chr4	49093177	52661966	4.1	2	0.00205358	1.03634
DQ596206	chr4	49093177	52661966	4.1	2	0.00205358	1.03634
AK097814	chr1	1645990	1943347	3.8	2	0.0031059	0.930962
CALML6	chr1	1645990	1943347	3.8	2	0.0031059	0.930962
TMEM52	chr1	1645990	1943347	3.8	2	0.0031059	0.930962
C1orf222	chr1	1645990	1943347	3.8	2	0.0031059	0.930962
SLC35E2	chr1	1645990	1943347	3.8	2	0.0031059	0.930962
GNB1	chr1	1645990	1943347	3.8	2	0.0031059	0.930962
KIAA1751	chr1	1645990	1943347	3.8	2	0.0031059	0.930962
CDK11B	chr1	1645990	1943347	3.8	2	0.0031059	0.930962
NADK	chr1	1645990	1943347	3.8	2	0.0031059	0.930962
ACTR3BP5	chr10	38772322	42818239	3.6	2	0.00520024	0.853701
ANXA10	chr4	169076438	169077537	0	-2	6.00E-05	-6.1742

Supplementary Table 5. Most up- and downregulated genes of patients #1 and #7

Downregulated genes<br>* downregulated in both patients			
Patient 1		Patient 7	
Gene Name	Fold Change (log2)	Gene	Fold Change (log2)
HAGHL	-3.68	PLA2G7	-1.36
TIFAB	-3.69	ATP2A3	-1.36
IL27	-3.70	IL7R	-1.36
MS4A4E	-3.71	SNORA49	-1.36
CARD11	-3.71	ITGA4	-1.36
SRPK3	-3.71	INPP5D	-1.37
PPARG	-3.72	HLA-DQA1	-1.37
AVPR2	-3.72	MST1R	-1.38
LOC105379051	-3.74	SNORA36C	-1.39
ZBP1	-3.74	CYTH4	-1.39
BLID	-3.75	FUCA1	-1.39
CSNK1G2-AS1	-3.76	ARHGEF6	-1.39
ZNF366	-3.77	SAMSN1	-1.39
FBXL15	-3.77	PRRG3	-1.40
IL20RB	-3.77	GIMAP4	-1.40
TRPV3	-3.77	MYH14	-1.40
TAF6L	-3.79	JAK3	-1.40
IZUMO4	-3.79	FGD2	-1.40
LIME1	-3.79	LAPTM5	-1.41
POU5F1	-3.79	CCL2	-1.41
OASL	-3.79	LCK	-1.41
ODF3B	-3.80	SNORD17	-1.41
UCA1*	-3.80	GIMAP5	-1.42
XIRP2	-3.81	ARHGAP25	-1.42
ATG9B	-3.81	CPNE4	-1.42
PAQR6	-3.82	OAS1	-1.42
SLC26A1	-3.82	FCRL5	-1.43
PNPLA7	-3.82	SPN	-1.43
UNC5A	-3.83	DDX60	-1.43
PLD4	-3.84	STAT1	-1.44
PSTPIP1	-3.84	DOCK2	-1.44
NKG7	-3.84	FCER1G	-1.44
MTRNR2L2	-3.85	GBP2	-1.45
FCER2	-3.86	HLA-DPA1	-1.45
ZNF296	-3.86	STC1	-1.45
SAA1	-3.87	SPI1	-1.46
CR2	-3.88	IQGAP2	-1.46
FLT3LG	-3.89	RNU5D-1	-1.46
TSPOAP1-AS1	-3.89	C1QB	-1.47
PHOSPHO1	-3.89	LSP1	-1.47
CARMIL2	-3.90	TSPAN7	-1.47
APOB	-3.90	RASAL3	-1.47
PIK3CD-AS2	-3.90	PRKCB	-1.47
VWA2	-3.90	CD38	-1.48
MUC4	-3.91	HLA-DRA	-1.48
AMH	-3.91	ADAMDEC1	-1.48
EPHB6	-3.91	SCARNA13	-1.49
BRSK2	-3.92	GPRIN3	-1.49
LINGO3	-3.93	CORO1A	-1.50
DTX1	-3.94	CD3E	-1.50
TMPRSS9	-3.95	NCKAP1L	-1.51
PRF1	-3.95	SRGN	-1.51
CRACR2B	-3.96	CTSC	-1.51
TSPAN32	-3.97	PIK3AP1	-1.51
GMPR	-3.97	HLA-F	-1.52
LINC00612	-3.97	SNORA36A	-1.52
CD22	-3.97	PLEK	-1.52
SHANK2	-3.98	SP140	-1.53
CARD9	-4.01	CAPN8	-1.53
P2RY8	-4.02	JCHAIN	-1.53
FAM30A	-4.02	ITGB2	-1.53
SAA2	-4.03	LCP2	-1.54
VPS9D1	-4.03	MIR142*	-1.55
MIR3687-2	-4.04	TNC	-1.55
CD72	-4.05	GVINP1	-1.55
CD79A	-4.07	SNORA54	-1.55
APOBEC3A	-4.09	SAMHD1	-1.56
CACNA1I	-4.10	BANK1	-1.57
JAKMIP1	-4.10	IFIT3	-1.57
MIR3648-2	-4.12	SNORD66	-1.57

Upregulated genes<br>*upregulated in both patients			
Patient 1		Patient 7	
Gene	Fold Change (log2)	Gene	Fold Change (log2)
TFPI2	5.43	CCDC80	1.74
CXCL14	5.42	COL11A1*	1.71
COL11A1*	5.42	ZFHX4	1.71
MEG3*	5.30	MEG3*	1.67
PTGS2	5.29	HTRA3*	1.66
MXRA5	5.06	COL6A6	1.59
IGFBP5	5.02	MAPK4	1.58
MMP11	4.95	COL8A1	1.57
THBS2*	4.94	ADAMTS17	1.57
PXDN	4.94	SLC2A3	1.57
RUNX1T1	4.93	PLOD2	1.53
FAM227A	4.85	COL1A1	1.52
MMP1	4.82	MBOAT2	1.51
SULF1	4.79	MMP2	1.50
PDGFRA	4.74	POU6F1	1.50
DIO2	4.71	ZIC5	1.48
COL12A1	4.67	CLDN3	1.47
HMCN1	4.64	PPP1R9A	1.46
HOXD1	4.62	P2RX2	1.46
TRPM8	4.62	AFAP1	1.46
HEY1	4.61	DNAH14	1.45
PDLIM3	4.59	PRG4	1.45
HOPX	4.58	PROSER2	1.45
DDIT4L	4.57	CBS	1.42
TMPRSS4	4.55	MEIS2	1.42
MAGI2-AS3	4.54	CCDC8	1.42
STC1	4.52	TRHDE-AS1	1.41
PDPN	4.52	PRKAA2	1.40
CTSE	4.49	COL1A2*	1.39
MMP13	4.49	RNF144A	1.39
CACHD1	4.48	TSPYL5	1.38
ADAMTS12	4.47	LAMA1	1.38
SLC7A2	4.42	CYP26B1	1.38
PRODH	4.42	HCAR2	1.37
MPDZ	4.41	PCLO	1.37
POSTN	4.38	LINC00641	1.37
ABCC9	4.37	ACOX3	1.37
CLDN2	4.36	COL9A3	1.36
LAMA4	4.35	AFAP1-AS1	1.36
IBSP	4.34	SLIT3	1.36
TSPAN12	4.33	SLIT2	1.36
CTGF*	4.33	ESYT3	1.35
PI15	4.33	WDR87	1.35
MYBPH	4.30	LMTK3	1.34
COL6A5	4.30	ATRNL1	1.34
IGSF3	4.29	TDRP	1.33
ESM1	4.25	MYO3A	1.33
ADGRD1	4.25	TMEM117	1.33
HSPB8	4.25	THBS2*	1.32
ETFRF1	4.24	CCDC149	1.31
SGIP1	4.23	NRCAM	1.31
RGS16	4.22	PLA2G12A	1.30
SNORD113-8	4.21	CGNL1	1.30
GJA1	4.21	ATP7B	1.30
GGTLC1	4.20	PLAGL1	1.30
KCNJ8	4.20	PTPN3	1.30
COL1A2*	4.20	TCHH	1.30
SYT7	4.18	CTGF*	1.30
COL5A1	4.18	SNX7	1.29
PKHD1	4.17	PLEKHG3	1.29
PGM5	4.17	STARD13-AS	1.29
PRRX1	4.16	MAML3	1.28
HTRA3*	4.14	DCN	1.28
MAP1B	4.14	CTSV	1.28
FGF7	4.13	MARVELD1	1.28
COL15A1	4.13	CC2D2A	1.28
PDGFRB	4.13	ZNF185	1.27
CPXM1	4.13	SLC6A2	1.27
FREM2	4.11	COL3A1*	1.27
SHROOM4	4.09	GJB2	1.27

MIR205	-4.14	IFI6	-1.58
PTGER2	-4.15	IKZF3	-1.58
FCRL1	-4.16	ITGAX	-1.58
PLIN4	-4.16	CXCL10	-1.59
LY9	-4.17	TRIM22	-1.59
PTPRCAP	-4.19	C1QA	-1.60
FAM129C	-4.25	CCL21	-1.60
PRKCQ	-4.27	BST2	-1.61
HBB	-4.29	FYB	-1.62
MTRNR2L8	-4.30	SERPINA1	-1.62
FGA	-4.33	XAF1	-1.62
HKDC1	-4.34	SLA	-1.62
NAPSB	-4.37	CXCL13	-1.62
PLIN5	-4.38	MS4A1	-1.63
LIMD2	-4.38	PTPRC	-1.64
RASGRP2	-4.39	CYFIP2	-1.64
ALB	-4.43	RSAD2	-1.65
RP1	-4.43	RELN	-1.65
SCML4	-4.45	CTSS	-1.66
ORM1	-4.50	SNHG12	-1.66
PF4	-4.58	IFI44	-1.67
TUBB1	-4.59	OAS2	-1.67
ABCC2	-4.60	SPOCK2	-1.68
MIR142*	-4.62	SNORA71D	-1.69
CRP	-4.63	UCA1*	-1.69
RUFY4	-4.66	DNAJC6	-1.69
NCF1	-4.68	NLRC5	-1.70
IRX2	-4.71	LCP1	-1.70
HP	-4.96	SNORA55	-1.72
AZGP1	-5.21	ITGAL	-1.79

LINC01021	4.08	DOCK3	1.26
UNC5C	4.08	FAM149A	1.26
RASSF8	4.07	MARK1	1.26
ITGA11	4.06	PNMA5	1.25
LONRF2	4.04	IQCA1	1.25
PRR26	4.03	SLC18A3	1.25
COL3A1*	4.03	SFRP2	1.24
SCIN	4.03	DGKH	1.22
KCNJ12	4.02	C2orf61	1.22
GPC6	4.02	PLSCR4	1.22
ADAMTS1	4.02	CHN2	1.22
GLI3	4.02	MIR30B	1.21
ACTG2	4.01	STXBP6	1.21
EGFL6	4.01	PQLC3	1.21
SNORD114-1	4.01	ALDH1A2	1.21
PPP1R1B	3.99	LINC00442	1.21
CXCL5	3.98	PLEKHA5	1.21
FBN1	3.98	PODN	1.21
PAEP	3.98	PALM2	1.21
LUM	3.98	PTPN13	1.21
KCNE4	3.96	PCDHGA2	1.20
DLC1	3.96	DLGAP2	1.20
CLIC6	3.96	MET	1.20
PTHLH	3.95	KIF26B	1.19
PTK7	3.95	KIAA1324	1.19
LOC101929705	3.94	FKBP10	1.19
RCAN2	3.94	CTHRC1	1.19
FAP	3.93	COL4A5	1.19
BICC1	3.93	KL	1.18
LAPTM4B	3.92	MISP	1.18

Supplementary Table 6. GO terms of the 100 most up- and downregulated genes in patient #1 and #7

Downregulated genes (GO terms)

Patient 1

Gene Set Name	# Genes in Gene Set	# Genes in Overlap	p-value	FDR q-value
GOBP_DEFENSE_RESPONSE	1816	27	6.39E-15	6.75E-11
GOBP_INFLAMMATORY_RESPONSE	850	19	1.06E-13	5.62E-10
GOBP_IMMUNE_RESPONSE	1940	24	1.32E-11	4.66E-08
GOBP_BIOLOGICAL_PROCESS_INVOLVED_IN_INTERSPECIES_ INTERACTION_BETWEEN_ORGANISMS	1722	22	6.03E-11	1.59E-07
GOBP_DEFENSE_RESPONSE_TO_OTHER_ORGANISM	1178	18	2.57E-10	5.43E-07
GOBP_CELL_ACTIVATION	1150	17	1.41E-09	2.49E-06
GOBP_REGULATION_OF_IMMUNE_SYSTEM_PROCESS	1523	19	2.21E-09	3.34E-06
GOBP_REGULATION_OF_IMMUNE_RESPONSE	918	15	3.84E-09	5.07E-06
GOCC_INTRINSIC_COMPONENT_OF_PLASMA_MEMBRANE	1795	19	3.10E-08	3.45E-05
GOBP_INNATE_IMMUNE_RESPONSE	921	14	3.27E-08	3.45E-05

Patient 7

Gene Set Name	# Genes in Gene Set	# Genes in Overlap	p-value	FDR q-value
GOBP_IMMUNE_RESPONSE	1940	53	2.84E-43	3.00E-39
GOBP_REGULATION_OF_IMMUNE_SYSTEM_PROCESS	1523	45	1.97E-37	1.04E-33
GOBP_POSITIVE_REGULATION_OF_IMMUNE_SYSTEM_PROCESS	966	37	2.47E-34	8.69E-31
GOBP_CELL_ACTIVATION	1150	38	6.11E-33	1.61E-29
GOBP_DEFENSE_RESPONSE	1816	43	1.23E-31	2.59E-28
GOBP_LYMPHOCYTE_ACTIVATION	841	31	4.70E-28	8.27E-25
GOBP_REGULATION_OF_IMMUNE_RESPONSE	918	31	6.55E-27	9.88E-24
GOBP_BIOLOGICAL_PROCESS_INVOLVED_IN_INTERSPECIES_INTERACTION_ BETWEEN_ORGANISMS	1722	37	2.04E-25	2.69E-22
GOBP_DEFENSE_RESPONSE_TO_OTHER_ORGANISM	1178	32	6.72E-25	7.88E-22
GOBP_REGULATION_OF_CELL_ACTIVATION	720	27	1.41E-24	1.49E-21

Upregulated genes (GO terms)

Patient 1

Gene Set Name	# Genes in Gene Set	# Genes in Overlap	p-value	FDR q-value
GOBP_EXTERNAL_ENCAPSULATING_STRUCTURE_ORGANIZATION	324	23	2.88E-27	3.04E-23
GOCC_EXTERNAL_ENCAPSULATING_STRUCTURE	575	26	9.93E-26	5.24E-22
GOCC_COLLAGEN_CONTAINING_EXTRACELLULAR_MATRIX	434	21	1.92E-21	6.75E-18
GOBP_TISSUE_DEVELOPMENT	1998	35	4.35E-21	9.15E-18
GOMF_EXTRACELLULAR_MATRIX_STRUCTURAL_CONSTITUENT	173	16	4.89E-21	9.15E-18
GOBP_CIRCULATORY_SYSTEM_DEVELOPMENT	1211	29	5.20E-21	9.15E-18
GOBP_CELL_ADHESION	1541	28	3.62E-17	5.45E-14
GOBP_TUBE_DEVELOPMENT	1155	24	5.09E-16	6.72E-13
GOBP_ANIMAL_ORGAN_MORPHOGENESIS	1058	23	8.73E-16	1.02E-12
GOBP_ANATOMICAL_STRUCTURE_FORMATION_INVOLVED_ IN_MORPHOGENESIS	1244	24	2.61E-15	2.76E-12

Patient 7

Gene Set Name	# Genes in Gene Set	# Genes in Overlap	p-value	FDR q-value
GOCC_COLLAGEN_CONTAINING_EXTRACELLULAR_MATRIX	434	19	9.76E-19	1.03E-14
GOMF_EXTRACELLULAR_MATRIX_STRUCTURAL_CONSTITUENT	173	14	9.20E-18	3.35E-14
GOCC_EXTERNAL_ENCAPSULATING_STRUCTURE	575	20	9.52E-18	3.35E-14
GOBP_EXTERNAL_ENCAPSULATING_STRUCTURE_ORGANIZATION	324	16	9.93E-17	2.62E-13
GOMF_EXTRACELLULAR_MATRIX_STRUCTURAL_CONSTITUENT_ CONFERRING_TENSILE_STRENGTH	41	8	7.72E-14	1.63E-10
GOCC_COLLAGEN_TRIMER	86	9	7.56E-13	1.33E-09
GOBP_ANIMAL_ORGAN_MORPHOGENESIS	1058	20	8.92E-13	1.35E-09
GOBP_TISSUE_DEVELOPMENT	1998	25	7.40E-12	9.77E-09
GOMF_STRUCTURAL_MOLECULE_ACTIVITY	808	17	1.05E-11	1.23E-08
GOCC_COMPLEX_OF_COLLAGEN_TRIMERS	22	6	1.23E-11	1.30E-08

Supplementary Table 7. Antibodies used for IMC

Metal Tag	Target	Antibody Clone	Lot	Host	Clonality	Vendor	Catalogue number	Final Concentration
Nd150	CD204	J5HTR3	4338161	Mouse	Monoclonal	eBioscience	14-9054-82	5 ug/mL
Tm169	VISTA	D1L2G	2	Rabbit	Monoclonal	Cell Signal Technologies	64953	3 ug/mL
Pr141	pan Cytokeratin	AE1	2341224	Mouse	Monoclonal	Milipore	MAB1612	2 ug/mL
Pr141	Keratin Epithelial	AE3	2607604	Mouse	Monoclonal	Milipore	MAB1611	2 ug/mL
In113	Histone H3	D1H2	15	Rabbit	Monoclonal	Cell Signal Technologies	4499BF	1 ug/mL
Nd142	CD38	EPR4106	GR3251769-4	Rabbit	Monoclonal	Abcam	ab176886	3 ug/mL
Sm149	CD11b	SP330	GR3258740-1	Rabbit	Monoclonal	Abcam	ab241408	4 ug/mL
Eu151	Indoleamine 2-3-dioxygenase (IDO)	SP260	GR3259345-2	Rabbit	Monoclonal	Abcam	ab245737	5 ug/mL
Yb172	CD31	EPR3094	GR3229164-2	Rabbit	Monoclonal	Abcam	ab207090	4 ug/mL
Yb173	CD14	SP192	GR3245776-1	Rabbit	Monoclonal	Abcam	ab230903	2 ug/mL
Y89	HLA-ABC	EMR8-5	565292	Mouse	Monoclonal	BD Biosciences	565292	4 ug/mL
Gd158	CD134	Ber-ACT35 (ACT35)	B247739	Mouse	Monoclonal	BioLegend	350002	2.5 ug/mL
Ho165	CD8a	C8/144B	2003078	Mouse	Monoclonal	EBioscience	14-0085-82	2 ug/mL
Yb172	vWF	poly vwf	3239865	Rabbit	Polyclonal	Milipore	AB7356	4 ug/mL
In115	SMA	1A4	2033287	Mouse	Monoclonal	Ebioscience	14-9760-82	0.25 ug/mL
Nd146	CD68	KP1	2071775	Mouse	Monoclonal	EBioscience	14-0688-82	3 ug/mL
Dy161	TCF1/TCF7	C63D9	9	Rabbit	Monoclonal	Cell Signal Technologies	2203	4 ug/mL
Nd143	HLA-DR	TAL 1B5	GR3222279-4	Mouse	Monoclonal	Abcam	ab20181	1 ug/mL
Lu175	CD206 (MMR)	685645	CEZR0217061	Mouse	Monoclonal	R&D Systems	MAB25341	4.5 ug/mL
Gd156	CD73	D7F9A	2	Rabbit	Monoclonal	Cell Signal Technologies	13160BF	4 ug/mL
Yb176	Cleaved Caspase3	C92-605	6074683	Rabbit	Monoclonal	Bectin Dickinson	559565	3 ug/mL
Bi209	CD15	HI98	B265372	Mouse	Monoclonal	BioLegend	301902	5 ug/mL
Dy163	FOXP3	236A/E7	2049450	Mouse	Monoclonal	EBioscience	14-4777-82	5 ug/mL
Er166	Carbonic Anhydrase IX	polyclonal_CA9_AF2188	VNQ0218041	Goat	Polyclonal	R&D Systems	AF2188	3 ug/mL
Er167	GITR / TNFRSF18	D5V7P	2	Rabbit	Monoclonal	Cell Signal Technologies	10419	10 ug/mL
Sm147	CD163	EDHu-1	149022a	Mouse	Monoclonal	Novus Biologicals	NB110-40686	4.5 ug/mL
Gd155	CD279 (PD-1)	D4W2J	4	Rabbit	Monoclonal	Cell Signal Technologies	86163BF	5 ug/mL
Sm152	CD3	polyclonal_A0452	20068606	Rabbit	Polyclonal	Dako	20068606	6 ug/mL
Yb171	CD4	EPR6855	GR3215375-27	Rabbit	Monoclonal	Abcam	ab181724	5 ug/mL
Eu153	LAG-3	D2G4O	5	Rabbit	Monoclonal	Cell Signal Technologies	15372BF	10 ug/mL
Tb159	Granzyme B	D6E9W	3	Rabbit	Monoclonal	Cell Signal Technologies	46890BF	5 ug/mL
Nd145	CD27	Polyclonal_CD27 / TNFRSF7	AXS0418031	Goat	Polyclonal	R&D Systems	AF382	10 ug/mL
Nd148	Siglec-1 / CD169	SP213	GR3259343-1	Rabbit	Monoclonal	Abcam	ab245735	5 ug/mL
Sm154	CD45RO	UCHL1	95787-13291511	Mouse	Monoclonal	Biolegend	304202	3 ug/mL
Dy162	CD45	2B11	2069169	Mouse	Monoclonal	eBioscience	14-9457-82	4 ug/mL
Er170	TMEM173	SP339	GR3245429-1	Rabbit	Monoclonal	Abcam	ab238796	4 ug/mL
Yb174	CCL21 / 6Ckine	Polyclonal_CCL21 / 6Ckine	AYJ218071	Goat	Polyclonal	R&D Systems	AF366	5 ug/mL
Nd144	CD278 (ICOS)	D1K2T	4	Rabbit	Monoclonal	Cell Signal Technologies	89601BF	5 ug/mL
Dy164	CD45RA	HI100	95783-13291513	Mouse	Monoclonal	BioLegend	304102	5 ug/mL

Gd160	CD274 (B7-H1-PD-L1)	73-10	GR3237208-3	Rabbit	Monoclonal	Abcam	ab226766	5 ug/mL
Er168	CD303	Polyclonal_DLEC / CLEC4C / BDCA-2	IDL0218101	Goat	Polyclonal	R&D Systems	AF1376	3 ug/mL

Supplementary Table 8. Antibodies used for immunohistochemistry

Target	Antibody Clone	Vendor	Catalogue number	Detection
PDL1	E1L3N	Cell Signaling	1368S	OptiView Kit Ventana
CD8	C8/144B	DAKO/Agilent	M7103	OptiView Kit Ventana
CD3	LN10	Leica/Biosystem	NCL-L-CD3-565	OptiView Kit Ventana
TIGIT	TG1	Dianova	DIA-TG1	HRP Refine Kit Leica
Tim3	4C4G3	Proteintech	60355-1-Ig	OptiView Kit Ventana
