# Supplementary material for: Acquired resistance to anti-PD1 therapy in patients with NSCLC associates with immunosuppressive T cell phenotype
Source: Nat Commun. 2023 Aug 24;14:5154. doi: 10.1038/s41467-023-40745-5 (PMC10449840; doi:10.1038/s41467-023-40745-5)
Supplement: Supplementary file 2 — Description of Additional Supplementary Files [file 41467_2023_40745_MOESM2_ESM.pdf]

Description of Additional Supplementary Files

File Name: Supplementary Data 1
Description: Genes with short variants of patient 1 only present at resistance

File Name: Supplementary Data 2
Description: Genes with copy number alterations (from WGS) in sample from tumor from patient 1 isolated at resistance
