# Supplementary material for: Acquired resistance to anti-PD1 therapy in patients with NSCLC associates with immunosuppressive T cell phenotype
Source: Nat Commun. 2023 Aug 24;14:5154. doi: 10.1038/s41467-023-40745-5 (PMC10449840; doi:10.1038/s41467-023-40745-5)
Supplement: Supplementary file 5 — Reporting Summary [file 41467_2023_40745_MOESM5_ESM.pdf]

nature portfolio

Corresponding author(s): A. Curioni-Fontecedro

Last updated by author(s): Jul 21, 2023

Reporting Summary

Nature Portfolio wishes to improve the reproducibility of the work that we publish. This form provides structure for consistency and transparency in reporting. For further information on Nature Portfolio policies, see our Editorial Policies and the Editorial Policy Checklist.

Statistics

For all statistical analyses, confirm that the following items are present in the figure legend, table legend, main text, or Methods section.

n/a	Confirmed	
☐	☒	The exact sample size (*n*) for each experimental group/condition, given as a discrete number and unit of measurement
☐	☒	A statement on whether measurements were taken from distinct samples or whether the same sample was measured repeatedly
☒	☐	The statistical test(s) used AND whether they are one- or two-sided<br>*Only common tests should be described solely by name; describe more complex techniques in the Methods section.*
☒	☐	A description of all covariates tested
☒	☐	A description of any assumptions or corrections, such as tests of normality and adjustment for multiple comparisons
☒	☐	A full description of the statistical parameters including central tendency (e.g. means) or other basic estimates (e.g. regression coefficient) AND variation (e.g. standard deviation) or associated estimates of uncertainty (e.g. confidence intervals)
☒	☐	For null hypothesis testing, the test statistic (e.g. *F*, *t*, *r*) with confidence intervals, effect sizes, degrees of freedom and *P* value noted<br>*Give P values as exact values whenever suitable.*
☒	☐	For Bayesian analysis, information on the choice of priors and Markov chain Monte Carlo settings
☒	☐	For hierarchical and complex designs, identification of the appropriate level for tests and full reporting of outcomes
☒	☐	Estimates of effect sizes (e.g. Cohen's *d*, Pearson's *r*), indicating how they were calculated

Our web collection on statistics for biologists contains articles on many of the points above.

Software and code

Policy information about availability of computer code

Data collection	Qupath v 0.2.3 for immunohistochemistry, Tiff files were generated from the IMC raw data using a customized python script
Data analysis	All custom code used for the IMC data in this study is available in the linked github repository. Tiff files were generated from the IMC raw data using a customized python script before segmenting cells using Ilastik pixel classification based on nuclear and membrane staining. Cell masks were created based on the ilastik probability maps using CellProfiler. Mean intensities for every marker were calculated for every cell. The raw counts were arcsinh transformed using cofactor 1. The single cell data was ultimately analyzed using R v3.6. Clustering was preformed using the PhenoGraph algorithm. The IMC data generated in this study and the R code have been deposited in the Zenodo database under the link https://zenodo.org/deposit/8041882. The genomic analysis and software versions used are available on Github https://github.com/Lab-Curioni/Acquired_Resistance_ICI_NSCLC.

For manuscripts utilizing custom algorithms or software that are central to the research but not yet described in published literature, software must be made available to editors and reviewers. We strongly encourage code deposition in a community repository (e.g. GitHub). See the Nature Portfolio guidelines for submitting code & software for further information.

Data

Policy information about availability of data

All manuscripts must include a data availability statement. This statement should provide the following information, where applicable:

- Accession codes, unique identifiers, or web links for publicly available datasets
- A description of any restrictions on data availability
- For clinical datasets or third party data, please ensure that the statement adheres to our policy

The raw sequencing data from the whole exome sequencing and RNA sequencing are available upon request (due to privacy reasons as sequencing might lead to identification of individuals). The code from the IMC analysis was developed in house and can be accessed through the public library GitHub, all image and metadata can be accessed on Zenodo https://zenodo.org/deposit/8041882. For genomic analysis the code and software versions used are available on Github https://github.com/Lab-Curioni/Acquired_Resistance_ICI_NSCLC.
Following databases were used for analysis:
GSVA MSigDB: Link: https://data.broadinstitute.org/gsea-msigdb/msigdb/release/6.2/h.all.v6.2.symbols.gmt, Version: GSEA MSigDB Release 6.2
WES& WGS: Human reference ucsc.hg19.fasta: Link: gs://gatk-legacy-bundles, Version: Gatk bundle 2.8
RNASeq: Human reference fasta, gff3 and gtf: Link: https://www.gencodegenes.org/human/release_19.html, Version: release 19
Cohort comparison: TCGA_LUAD_rnaseqV2_RSEM_genes_normalized, Link: https://gdac.broadinstitute.org/, Version: V2

Human research participants

Policy information about studies involving human research participants and Sex and Gender in Research.

Reporting on sex and gender	Sex (biological attribute) has been indicated
Population characteristics	The population characteristics can be found in Table 1.
Recruitment	Patients with NSCLC with acquired resistance to immune checkpoint blockade treated at the University Hospital Zurich (see methods). Patients were selected according to the availability and quality of tumor material before start of treatment and at acquired resistance.
Ethics oversight	The study was approved by the Cantonal Ethical Committee Zurich (KEK-ZH-2018-01919, KEK-ZH-2020-02566). All patients included in the study provided informed written consent. The study was performed in accordance with the declaration of Helsinki.

Note that full information on the approval of the study protocol must also be provided in the manuscript.

Field-specific reporting

Please select the one below that is the best fit for your research. If you are not sure, read the appropriate sections before making your selection.

☒ Life sciences ☐ Behavioural & social sciences ☐ Ecological, evolutionary & environmental sciences

For a reference copy of the document with all sections, see nature.com/documents/nr-reporting-summary-flat.pdf

Life sciences study design

All studies must disclose on these points even when the disclosure is negative.

Sample size	Not applicable, all patients with NSCLC with acquired resistance who had enough material for analysis at response and at resistance were included. No sample size calculation have been performed, as well as no statistical analysis: this is an intra-patient analysis where samples from the same patient are compared before and at resistance to a treatment.
Data exclusions	No data exclusion
Replication	No replications have been performed as we analysed one sample/timepoint per patient.
Randomization	This is an intra-patient analysis where samples from the same patient are compared before and at resistance to a treatment, no radomization is applicable
Blinding	This is an intra-patient analysis where samples from the same patient are compared before and at resistance to a treatment, no blinding is applicable

Reporting for specific materials, systems and methods

We require information from authors about some types of materials, experimental systems and methods used in many studies. Here, indicate whether each material, system or method listed is relevant to your study. If you are not sure if a list item applies to your research, read the appropriate section before selecting a response.

Materials & experimental systems

n/a	Involved in the study	
☐	☒	Antibodies
☒	☐	Eukaryotic cell lines
☒	☐	Palaeontology and archaeology
☒	☐	Animals and other organisms
☐	☒	Clinical data
☒	☐	Dual use research of concern

Methods

n/a	Involved in the study	
☒	☐	ChIP-seq
☒	☐	Flow cytometry
☒	☐	MRI-based neuroimaging

Antibodies

Antibodies used	see Table 9, supplementary information
Validation	All antibodies used in this study are validated for their use on FFPE tissues by the manufacturers. All antibodies have been assigned an RRID and can be linked to other publications. Each antibody is further tested in our lab for its tissue specificity in the tissue used, using both fluorescence microscopy and imaging mass cytometry imaging with co-staining of other markers

Clinical data

Policy information about clinical studies
All manuscripts should comply with the ICMJE guidelines for publication of clinical research and a completed CONSORT checklist must be included with all submissions.

Clinical trial registration	not applicable
Study protocol	not applicable
Data collection	Clinical data were collected from the local hospital patient information system
Outcomes	Clinical data were collected from the local hospital patient information system
